# Trawl ban in a heavily exploited marine environment: Responses in population dynamics of four stomatopod species

**DOI:** 10.1038/s41598-018-35804-7

**Published:** 2018-12-14

**Authors:** Lily S. R. Tao, Karen K. Y. Lui, Edward T. C. Lau, Kevin K. Y. Ho, Yanny K. Y. Mak, Yvonne Sadovy de Mitcheson, Kenneth M. Y. Leung

**Affiliations:** The Swire Institute of Marine Science and School of Biological Sciences, The University of Hong Kong, Pokfulam, Hong Kong China

## Abstract

Intensive trawling activities in Hong Kong waters have seriously depleted fishery resources and damaged marine benthic habitats over the last four decades. To minimize further destruction and rehabilitate fishery resources, the Hong Kong Government implemented a permanent territory-wide trawling closure on 31 December 2012. Such a trawl ban creates a unique opportunity to investigate recoveries in ecosystem structure and function following a major shift in disturbance regime by removing impacts from a major gear. This study was designed to test the hypothesis that dominant predatory mantis shrimps, including *Harpiosquilla harpax*, *Miyakella nepa*, *Oratosquillina interrupta*, and *Oratosquilla oratoria* would show signs of recovery following the trawl ban. Their population dynamics were investigated before and after the trawl ban. The results showed that their mean weight, mean carapace length and proportion of large-sized individuals increased significantly 3.5 years after the trawl ban, whilst their abundance, biomass and maximum length remained unchanged. This study suggests that the stomatopod assemblage in the human-dominated Hong Kong waters shows some initial signs of possible recovery following the trawl ban but also highlights the complexity of implementing fishery management and detecting changes resulted from management measures in a heavily urbanized seascape where many biotic and abiotic factors can influence their population dynamics.

## Introduction

Hong Kong has transformed from a small fishing port with about 7,500 Chinese people in 1841 to an international metropolis with a population of over 7.3 million in 2017^1,2^. Notably, Hong Kong had a vibrant fisheries industry between the mid-1950s and the late 1970s^3–5^. Owing to growing market demand driven by an increasing population and government-subsidized mechanization of fishing vessels, in the absence of fisheries management local marine resources were over-exploited by an array of fishing methods (i.e., bottom and pelagic trawls, gillnets, traps, purse seines, and hook and line) since the late 1970s^5,6^. Among all fishing methods used, pair, stern, shrimp and hang trawlers accounted for 50% of the total fisheries landings in Hong Kong waters with an annual catch of ~12,000 tonnes in 2006^7^. Trawling is regarded as the most destructive fishing method because trawlers unselectively capture marine organisms, irrespective of their commercial or ecological values. In particular, bottom trawlers also seriously damage and disturb benthic habitats essential to benthic and demersal communities^8–10^.

To mitigate the problem of overfishing and the adverse effects of trawling, the Government of the Hong Kong Special Administrative Region imposed a permanent territory-wide ban on pair, stern, shrimp and hang trawling over an area of about 1,700 km^2^ of Hong Kong’s territorial waters on 31 December 2012^11^. This ban is also intended to rehabilitate the marine benthic ecosystem and associated marine biodiversity and fisheries resources in Hong Kong waters.

Similar trawl ban policies have been adopted in other parts of the world with varying degrees of success. Increases in abundance and biomass of several commercial species were reported on the Scotian Shelf of Canada and in the Gulf of Castellammare of Italy after the introduction of trawl bans^12–14^. Implementing restrictive management brought notable increases in catches in Indonesia^15,16^ and Kenya^17^ within two years of introduction of trawl bans, which stimulated further heavy exploitation by other legal, non-trawling fishing methods and resulted in further declines in catches^15,18^. Such variable outcomes of a trawl ban are likely ascribable to the uniqueness of each ecosystem, as well as displacement and unintended consequences of and responses to such management intervention in different countries due to social and economic factors. Another factor is the effectiveness of enforcement.

For an individual species, increases in abundance, biomass and size are commonly used to indicate recovery after management interventions such as trawl bans or no-take zones (NTZ)^19–22^. For example, the annual production of sea scallops (*Placopecten magellanicus*) and green sea urchins (*Strongylocentrotus droebachiensis*) showed increases after six years’ closure in Georges Bank, Southern New England^20^. Signs of recovery were also detected in bivalves at the Offshore Wind farm Egmond aan Zee (OWEZ) in the North Sea where higher abundance of *Spisula solida* and larger size of *Tellina fabula* and *Ensis directus* were recorded after four years of exclusion of trawling activities^22^. A similar finding was also noted in the Gulf of Castellammare where an increase in the size of the anglerfish *Lophius budegassa* was recorded nine years after the trawl ban^19^.

Life history traits exert strong influence on a species’ potential to recover after physical disturbances, such as trawling and biological overexploitation. Small, short-lived, fast growing species, such as algae and certain invertebrates, are expected to have higher potential and take shorter time to recover from overfishing and habitat disturbances^23–26^. Stomatopods (commonly known as mantis shrimps) are a group of short-lived (life span usually no more than four years) predatory invertebrates^27^, and hence are expected to show signs of improvement more quickly following management compared to other carnivorous crustaceans or to longer lived species. At the same time, stomatopods not only are economically important fishery resources worldwide^28,29^, they also play an important ecological role in structuring marine benthic food webs^30–33^. They form part of the diet of other predatory and scavenging crustaceans^31^, fishes^34,35^ and seabirds^36^, and compete with benthopelagic carnivorous fishes in tropical marine waters^32^. Moreover, as stomatopods build and live in burrows, they affect the energy flow and microbial-driven nutrient cycling through bioturbation of sediments^37–39^ and in turn depend on appropriate sediment characteristics for their burrows^40,41^. Owing to their economic and ecological importance, it is of interest to investigate whether the stomatopod assemblage in Hong Kong waters showed signs of recovery within a few years of the implementation of the trawl ban.

Previous studies on local stomatopods found that a higher abundance of this taxon inhabited the western waters of Hong Kong, accounting for around 83% of total stomatopod catches across Hong Kong by weight^27,42^. Four dominant stomatopod species, namely *Harpiosquilla harpax*, *Miyakella nepa*, *Oratosquillina interrupta*, and *Oratosquilla oratoria* with life spans of 1 to 2 years jointly contributed to about 46% of the total crustacean catch from Hong Kong waters and 98% of the total stomatopod catches in the western waters^27^.

This study, therefore, investigated the population structures of the four dominant stomatopod species in the western waters of Hong Kong before, immediately after and 3.5 years after the implementation of the trawl ban in 2012. To evaluate the effectiveness of the trawl ban, the abundance, biomass and three size-based indicators [i.e., mean individual weight, mean carapace length and maximum carapace length (L_95%_ represents the upper 95% quartile of the carapace length and is recommended as an alternative to maximum observed length (L_max_) and asymptotic length (L_∞_) which are strongly influenced by sample size^43^)] of the four species were determined^44^. Based on the assumptions that fishing pressure and physical disturbance on the benthic ecosystem would both be reduced after the trawl ban, this study aimed to test three hypotheses; that there would be increases in (1) abundance and biomass of the four dominant stomatopod species, (2) relative abundance and biomass of stomatopods as a proportion of total carnivorous crustaceans, considering both mantis shrimps and carnivorous crabs (some of them may have a relatively greater longevity of around three to four years)^45–47^ and (3) three size-based indicators and the proportion of large-sized individuals of the four stomatopod species.

## Results

### Environmental variables

Percentage mud content, suspended solids (SS), dissolved oxygen (DO), Chlorophyll-a concentration (Chl-a) and bottom water temperature (Temp) did not differ significantly among the three surveyed study time periods (Fig. S1a–e). However, pH in the western waters of Hong Kong decreased significantly from 8.12 in 2004 to 7.86 in 2015–2016 (Fig. S1f; One-way ANOVA: *F*_*2*,*9*_ = 14.9, *p* < 0.01).

### Population structure and recruitment period

Size frequency histograms for the four stomatopod species at western waters of Hong Kong during the three survey years are shown in Figs S2–S5 (in Supplementary Information). The number of cohorts of the four species in each surveyed month ranged from 0 to 4 among all three years (Figs S6–S9), suggesting variable spawning periods among and within the four species and varying recruitment periods in many months of the year. New cohorts of *Harpiosquilla harpax* appeared from August to September, indicating its recruitment period (Fig. S2). Recruits of *Miyakella nepa* were found in five consecutive months from August to December of each survey year (Fig. S3). *Oratosquillina interrupta* recruited mainly in July, August and October with some winter recruits in January and December (Fig. S4). Recruitment of *Oratosquilla oratoria* occurred in May and December of 2004, and in February, March, July and December in 2013–2014 and 2015–2016 (Fig. S5).

### Growth

The average growth rate in terms of carapace length for each stomatopod species ranged from 2.19 (*Oratosquilla oratoria* in 2004) to 4.82 (*H*. *harpax* in 2013–2014) mm per month (Table 1). Difference in growth rate was detected among years in population of *H*. *harpax* (One-way ANOVA: *F*_*2*,*12*_ = 5.73, *p* < 0.05), with the lowest growth rate found in 2015–2016. Growth rate of the other three stomatopod species did not show any yearly significant differences.

**Table 1:** Mean growth rate, cumulative number of cohort, Von Bertalanffy growth function (VBGF) growth parameters and Growth Performance Index (ϕ′) for four stomatopod species collected in the western waters of Hong Kong from January 2004 to December 2004 (2004; before the trawl-ban), June 2013 to May 2014 (2013–2014; immediately after the trawl-ban), and June 2015 to May 2016 (2015–2016; 3.5 years after the trawl-ban).

Year	Mean growth rate (SD) (mm/month)	Cumulative number of cohort	L_∞_ (mm)	K (/year)	ϕ′
**(a)** ***Harpiosquilla harpax***					
2004	4.31 (0.57)	6	47.25	0.39	2.94
2013–2014	4.82 (0.69)	6	53.55	0.39	3.05
2015–2016	3.06 (0.10)	3	49.35	0.46	3.05
**(b)** ***Miyakella nepa***					
2004	2.56 (0.90)	6	32.55	0.58	2.79
2013–2014	3.59 (1.91)	5	32.55	0.44	2.67
2015–2016	3.30 (2.20)	3	34.65	1.3	3.19
**(c)** ***Oratosquillina interrupta***					
2004	2.36 (0.83)	5	36.75	1	3.13
2013–2014	2.88 (0.76)	7	40.95	0.43	2.86
2015–2016	3.58 (0.23)	4	34.65	0.86	3.01
**(d)** ***Oratosquilla oratoria***					
2004	2.19 (1.33)	3	36.75	1.2	3.21
2013–2014	3.86 (1.86)	6	38.85	0.48	2.86
2015–2016	2.45 (0.53)	4	30.45	0.96	2.95

The estimated ELEFAN parameters, L_∞_, K and ϕ′ for the four stomatopod species differed among years (Table 1). The three parameters showed increasing trends for *H*. *harpax* after the trawl ban, but no general trend was observed for the other three species.

### Abundance and biomass

These four stomatopod species jointly contributed to 38.8–73.2% total abundance and 35.6–62.5% total biomass (Table 2) in terms of total commercial crustacean catches (Table S1) in the western waters of Hong Kong. The four species showed clear seasonal variations across all three study years, having higher abundance and biomass in August–October than in January–March (Fig. S10). The biomass and abundance of the four species were low during January–May of 2016 when compared with those of the same period in the other two years.

**Table 2:** Percentage of abundance/biomass (% over total mantis shrimp abundance/biomass) for each stomatopod species (*Oratosquilla oratoria*, *Oratosquillina interrupta*, *Miyakella nepa* and *Harpiosquilla harpax*) and percentage of total abundance/biomass of the four investigated mantis shrimps (% over total commercial crustacean abundance/biomass) at western waters from January 2004 to December 2004 (before the trawl-ban) and June 2013 to May 2014 (2013–2014; immediately after the trawl-ban), June 2015 to May 2016 (2015–2016; 3.5 years after the trawl-ban).

Year	2004	2013–2014	2015–2016
**(a) Abundance (%)**			
*Harpiosquilla harpax*	10.01	10.42	14.60
*Miyakella nepa*	15.28	22.00	25.40
*Oratosquillina interrupta*	40.60	27.64	47.91
*Oratosquilla oratoria*	34.11	39.94	12.09
4MS A/Total A	73.19	39.74	38.83
**(b) Biomass (%)**			
*Harpiosquilla harpax*	20.29	19.73	25.31
*Miyakella nepa*	14.93	16.22	24.33
*Oratosquillina interrupta*	39.78	29.91	41.44
*Oratosquilla oratoria*	25.00	34.14	8.92
4MS B/Total B	62.51	47.44	35.55

4MS A/B/Total A/B: percentage of total abundance/biomass of four investigated mantis shrimps over total commercial crustacean abundance/biomass.

The dominant stomatopod species varied among the years surveyed. *Oratosquillina interrupta* was the dominant species in 2004 and 2015–2016, contributing to 40.6% and 47.9% of total stomatopod abundance respectively, while *Oratosquilla oratoria* dominated in 2013–2014, accounting for 39.9% of total stomatopod abundance (Table 2a). Similar trends were also observed in terms of biomass (Table 2b).

**Figure 1:**
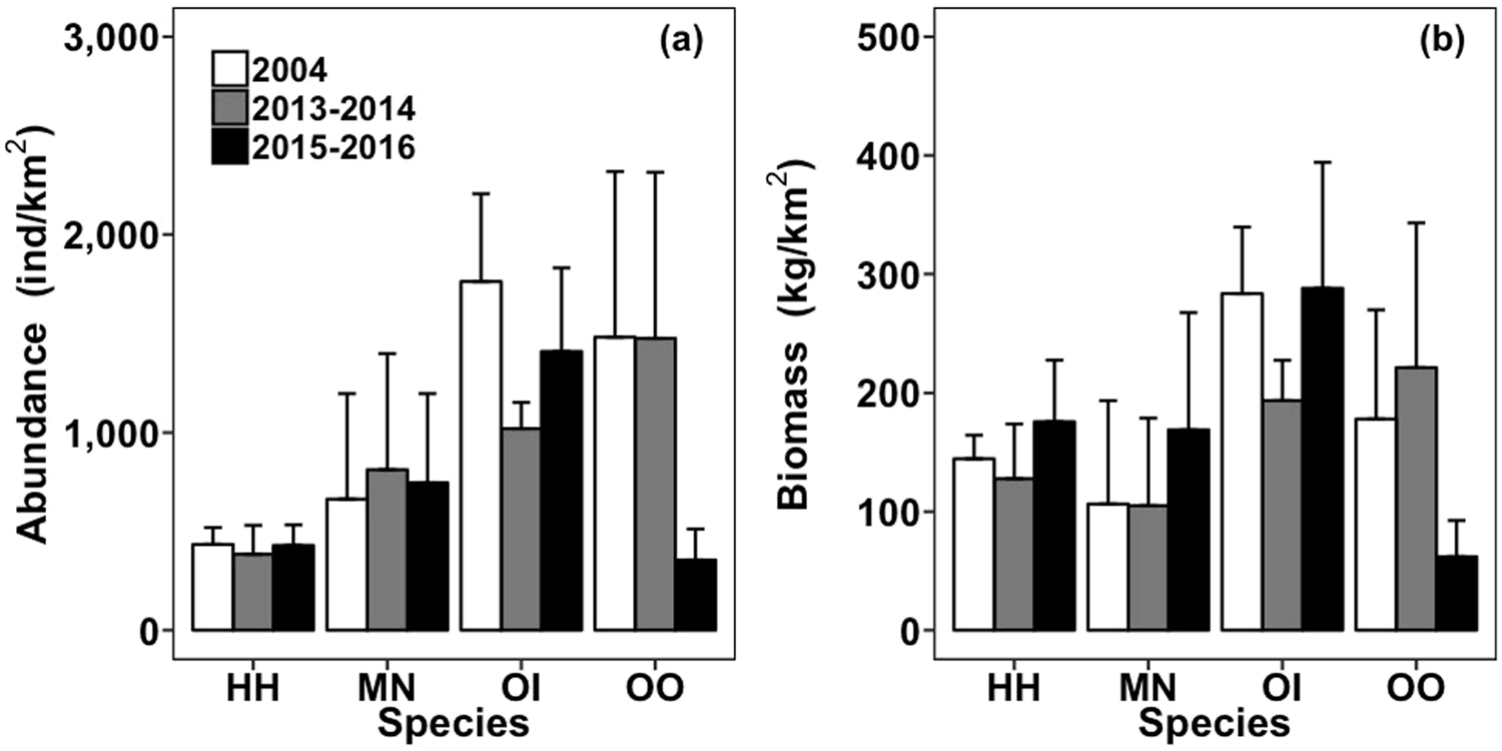
(**a**) Monthly abundance (mean + SE; individuals/km^2^) and (**b**) biomass (mean + SE; kg/km^2^) of four stomatopod species collected in the western waters of Hong Kong from January 2004 to December 2004 (2004; before the trawl ban), June 2013 to May 2014 (2013–2014; immediately after the trawl ban), and June 2015 to May 2016 (2015–2016; 3.5 years after the trawl ban). HH: *Harpiosquilla harpax*, MN: *Miyakella nepa*, OI: *Oratosquillina interrupta*, OO: *Oratosquilla oratoria*.

**Figure 2:**
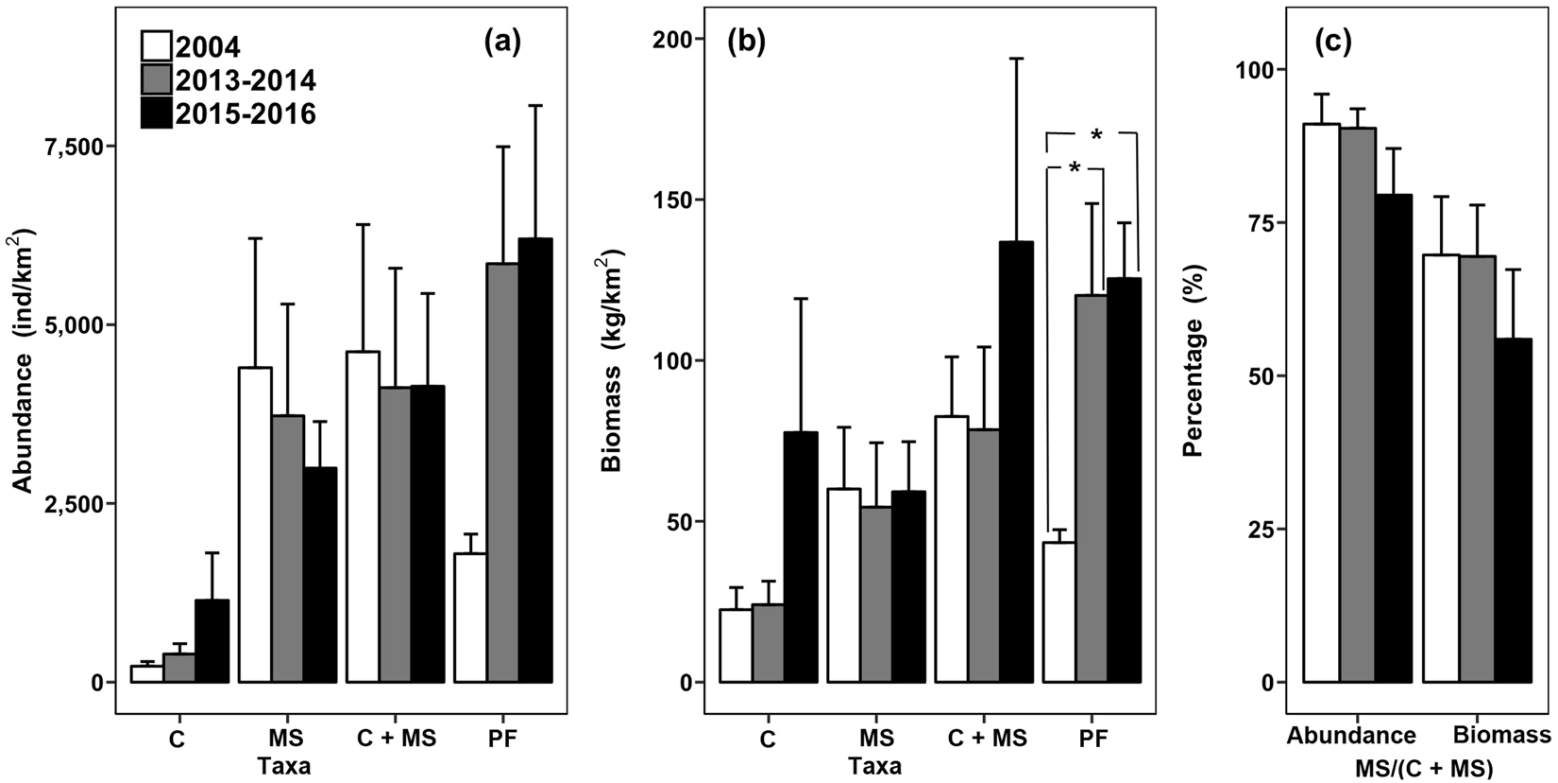
(**a**) Monthly abundance (mean + SE; individuals/km^2^), (**b**) biomass (mean + SE; kg/km^2^) for carnivorous crabs (C), four stomatopod species (MS), total carnivorous crustaceans (C + MS) and predatory fishes (PF), and (**c**) relative abundance and biomass of four stomatopod species over total carnivorous crustacean collected in the western waters of Hong Kong from January 2004 to December 2004 (2004; before the trawl ban) and June 2013 to May 2014 (2013–2014; immediately after the trawl ban) and June 2015 to May 2016 (2015–2016; 3.5 years after the trawl ban). Significant differences are indicated by asterisks: *p < 0.05; **p < 0.01, ***p < 0.001.

The abundance and biomass of the four study stomatopod species in western waters were highly variable within and between the surveyed years, but did not differ significantly among surveyed years (Fig. 1). Likewise, no significant temporal changes were detected in total abundance and biomass of carnivorous crabs, stomatopods or in total carnivorous crustaceans (Fig. 2a,b). However, the biomass of predatory fishes significantly increased after the trawl ban (*F*_*2*,*9*_ = 5.59, *p* < 0.05), while no such an increase was detected in their abundance (Fig. 2a,b). No significant yearly difference was observed in relative abundance and biomass of stomatopods (Fig. 2c). The results from the multiple regression analysis showed that temperature and pH had significant relationships with abundance and biomass for all stomatopods (Table S2).

### Size-based indicators

**Figure 3:**
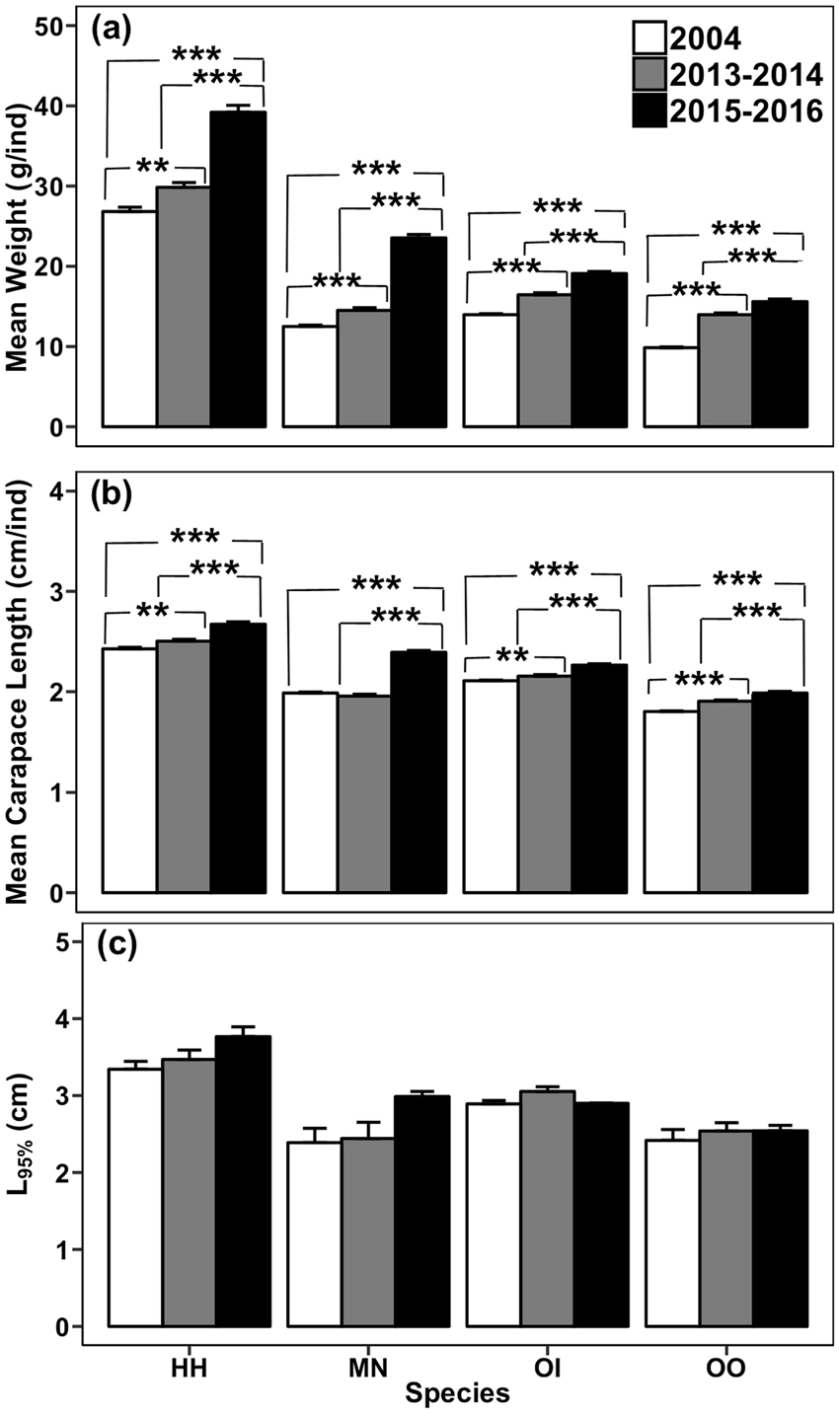
(**a**) Mean weight (mean + SE; g/individual), (**b**) mean carapace length (mean + SE; cm/individual), and (**c**) L_95%_ (mean + SE; cm) of the four stomatopod species collected in the western waters of Hong Kong from January 2004 to December 2004 (2004; before the trawl ban), June 2013 to May 2014 (2013–2014; immediately after the trawl ban), and June 2015 to May 2016 (2015–2016; 3.5 years after the trawl ban). HH: *Harpiosquilla harpax*, MN: *Miyakella nepa*, OI: *Oratosquillina interrupta*, OO: *Oratosquilla oratoria*. Significant differences are indicated by asterisks: *p < 0.05; **p < 0.01, ***p < 0.001.

Both mean weight and mean carapace length of the four stomatopod species increased significantly after the trawl ban in the survey area (Fig. 3a,b). From 2004 to 2016, the mean weight significantly increased by 46.1%, 88.3%, 36.7% and 58.4% for *H*. *harpax*, *M*. *nepa*, *Oratosquillina interrupta* and *Oratosquilla oratoria*, respectively (*F*_*2*,*3015*_ = 94.80, *p* < 0.001; *F*_*2*,*3827*_ = 391.19, *p* < 0.001; *F*_*2*,*8803*_ = 192.05, *p* < 0.001; *F*_*2*,*7300*_ = 335.01, *p < *0.001). The carapace length increased by 10.1%, 20.5%, 7.4% and 10.2% for *H*. *harpax*, *M*. *nepa*, *Oratosquillina interrupta* and *Oratosquilla oratoria*, respectively (*F*_*2*,*3015*_ = 45.727, *p* < 0.001; *F*_*2*,*3827*_ = 210.73, *p* < 0.001; *F*_*2*,*8803*_ = 53.662, *p* < 0.001; *F*_*2*,*7300*_ = 81.449, *p* < 0.001). No significant difference was observed in L_95%_ for the four species among years (Fig. 3c).

**Figure 4:**
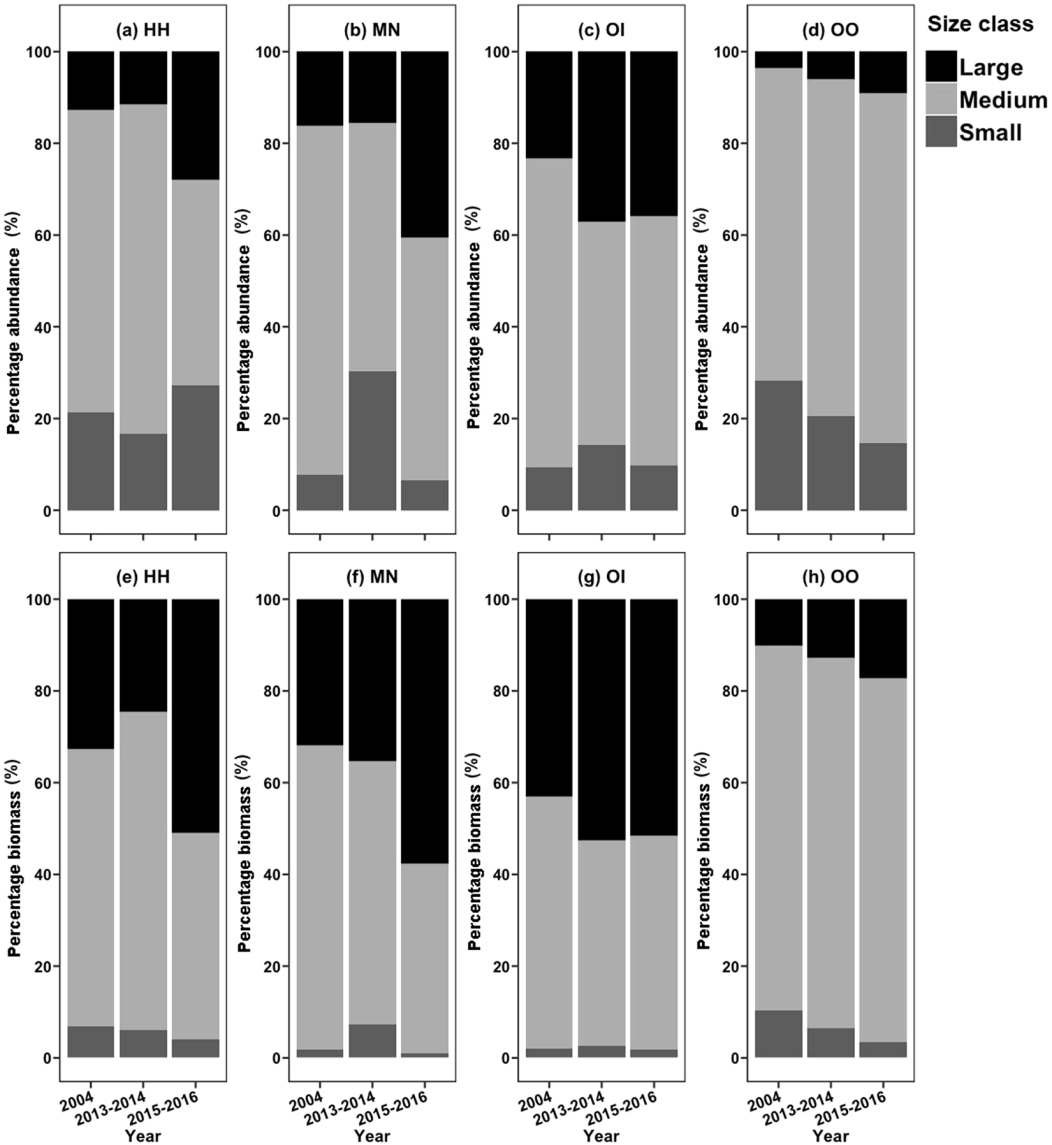
Yearly comparison (2004, 2013–2014 vs. 2015–2016) of percentage abundance (**a**–**d**) and biomass (**e**–**h**) of three size classes for four stomatopod species in the western waters of Hong Kong, respectively. HH: *Harpiosquilla harpax*, MN: *Miyakella nepa*, OI: *Oratosquillina interrupta*, OO: *Oratosquilla oratoria*.

Even though the proportion of large individuals of *H*. *harpax* remained similar before and immediately after the trawl ban, an increase was observed 3.5 years after the trawl ban (Fig. 4a,e). A similar trend was also observed in terms of absolute value of abundance and biomass in *H*. *harpax* (Fig. S11a,b). Increasing trends after the trawl ban were observed in percentage abundance of large size class in *Oratosquillina interrupta* and *Oratosquilla oratoria*, and in percentage biomass of large size class in *M*. *nepa*, *Oratosquillina interrupta* and *Oratosquilla oratoria* (Fig. 4b–d,f–h). However, increased absolute value of abundance and biomass in large size class were only observed in *M*. *nepa* following the trawl ban (Fig. S11c,d), whereas no such an increase was found in *Oratosquillina interrupta* and *Oratosquilla oratoria* (Fig. S11e–h).

### Size spectra of four stomatopods species

**Figure 5:**
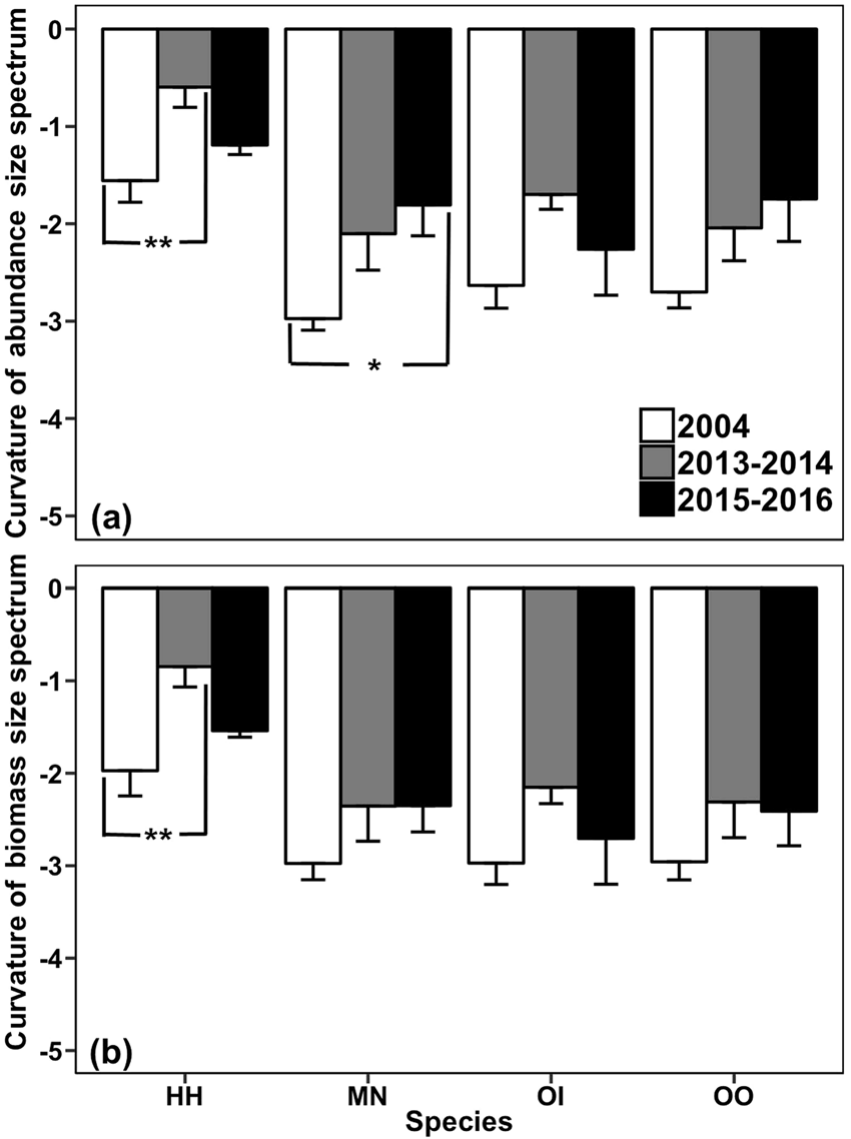
(**a**) The curvature of abundance (mean + SE) and (**b**) biomass (mean + SE) size spectrum at western waters from January 2004 to December 2004 (before the trawl ban) and June 2013 to May 2014 (2013–2014; immediately after the trawl ban), June 2015 to May 2016 (2015–2016; 3.5 years after the trawl ban). HH: *Harpiosquilla harpax*, MN: *Miyakella nepa*, OI: *Oratosquillina interrupta*, OO: *Oratosquilla oratoria*. Significant differences are indicated by asterisks: *p < 0.05; **p < 0.01.

The curvature derived from each species’ size spectrum showed variations among years (Fig. 5). The curvatures from abundance and biomass size spectra of *H*. *harpax* significantly increased immediately after the trawl ban when compared with those of 2004 (*F*_*2*,*24*_ = 7.084, *p* < 0.01; *F*_*2*,*24*_ = 7.928, *p* < 0.01; Fig. 5). Likewise, the curvature from the *M*. *nepa* abundance size spectrum significantly increased 3.5 years after the trawl ban when compared with that of 2004 (*F*_*2*,*17*_ = 3.839, *p* < 0.01; Fig. 5a). No significant temporal change in the curvature was observed for *Oratosquillina interrupta* and *Oratosquilla oratoria*.

## Discussion

The implementation of the trawl ban in Hong Kong waters was intended to reduce benthic physical disturbance and total fishing effort, and hence would contribute towards rehabilitating fisheries resources^14,48^. With removal of disturbance and/or reduction of exploitation, recovery is anticipated and likely to be measureable as some form of increase in terms of biological attributes and responses^26^. Responses at the population level are often measured in terms of species’ abundance^19,49^, biomass^13,14,49^, size structure^21,22,49^ and life history traits, such as size and age at maturity, maximum size, longevity, growth rate, and natural mortality etc.^23,50,51^. The present study adopted an integrated approach by measuring temporal changes (i.e., before, immediately after and 3.5 years after the trawl ban in Hong Kong) in environmental variables, and abundance, biomass and size structure of the four dominant stomatopod species in the western waters of Hong Kong.

In terms of environmental variables, the marine waters in western Hong Kong have become more acidic over the last decade. Other water properties and physical structure of sediment did not differ significantly over the study period. However, suspended solids showed a declining trend after the trawl ban which may reflect declines in physical disturbance on the benthic floor^52,53^.

In terms of changes in abundance, biomass and size structure of the four target stomatopods, results were variable. Increases in individual sizes, as reflected by greater weight and carapace length, were evident within 3.5 years of the introduction of the trawl ban in all four stomatopods. The proportion of large-sized individuals of all four species also increased 3.5 years after the trawl ban. However, no changes were observed in absolute and relative abundance and biomass, or in maximum body length, suggesting possible confounding factors hindering the anticipated recovery of these four relatively short-lived, stomatopod species, or otherwise influencing these parameters.

### Size-based indicators

Size-based indicators (i.e., mean length, mean weight and maximum length) are often applied to investigate the effects of fishing or disturbance to marine ecosystems^26,44^. In particular, they are considered to be sensitive to fishing impacts^43^. For example, Bergman *et al*.^22^ used an increase in mean shell length as an indicator of recovery in the bivalves *Tellina fabula* and *Ensis directus* in the OWEZ five years after the implementation of a ban on bottom trawling. Increases in mean carapace length of the legal-sized lobsters (*Homarus gammarus* L.) and mean carapace width of the brown crabs (*Cancer pagurus* L.) were also taken as an indication of recovery in the NTZ in an area adjacent to Lundy Island in the UK after four years of closure^21^. In the current study, we observed increases in mean weight and carapace length of four stomatopod species 3.5 years after the implementation of the trawl ban. Even though this management intervention had only been in place for a relatively short period, the observed increases in mean weight and carapace length of the stomatopods could have been a direct result of reduced physical disturbance on the benthic environment and/or of reduced fishing pressure due to a substantial decline in trawling activities after the trawl ban. For example, an undisturbed benthic substrate may be more suitable for burrow-building. We had similar findings in the crab assemblages in the Outer Estuary (WO; W3 and W4 in this study) of the western waters of Hong Kong which also showed significant increases in their mean weight and carapace length in 2015–2016 when compared with those of 2013–2014^54^ (*p* < 0.01; Fig. S12).

Size spectra have been widely used as tools to detect fishing pressure and responses to management in marine ecosystems^55–57^. A shallow slope or curvature (less negative value) in the size spectrum suggests a relatively higher abundance or biomass of large-sized individuals^43,58^. The present results were consistent with the above findings at the local ‘population’ (the biological extent of the population is not known) level. We found that the curvature from abundance and biomass size spectra of *Harpiosquilla harpax* significantly increased a few months after the trawl ban, with an increased percentage abundance of the medium size class (>2 and ≤3 cm; Fig. 4a) and especially in the large medium size class (>2.5 and ≤3 cm). The significant increase in the curvature of abundance size spectrum of *Miyakella nepa* 3.5 years after the trawl ban was attributable to an increased percentage abundance of large-sized class (Fig. 4b). Furthermore, a general increasing trend in the curvature of both abundance and biomass size spectra was found in the other two species, *Oratosquillina interrupta* and *Oratosquilla oratoria*, with greater proportions of large-sized animals (Fig. 4c,d,g,h). These findings were consistent with the anticipation of higher mean individual weight and mean carapace length after the trawl ban.

### Abundance and biomass indicators

The present study failed to detect any increases in either relative or absolute abundance or biomass among the four commercially important stomatopod species 3.5 years after the implementation of the trawl ban in Hong Kong. Several underlying factors, such as biological interactions, environmental variables, anthropogenic disturbances and study duration, may fully or partially account for the absence of increases in their abundance and biomass. Biological interactions, such as inter-species competition and predation from other fauna, impose high pressure on the four stomatopod species after the trawl ban that could mask, slow or prevent the anticipated recovery. For example, a decrease in the abundance of the velvet crab *Necora puber* was reported in NTZ of Lundy Island in the UK four years after the implementation of a closure, which could have been a result of predation and/or competition from the lobster *Homarus gammarus*^21^. The biomass of carnivorous crabs showed an increasing trend after the trawl ban in western waters (Fig. S13b), particularly in the Outer estuary (WO) area where significant increases were recorded^54^. Furthermore, the biomass of predatory fishes also increased significantly in the western waters of Hong Kong (Figs 2b and S13), which imposed high predatory pressure on larvae and juveniles of stomatopods, and led to strong competition with adult stomatopods.

The present study detected that pH and temperature could have had significant effects on abundance and biomass of stomatopods. Recent work has suggested that subtle changes in seawater acidity could have a negative effect on moulting frequency in marine crustaceans such that species’ survival and abundance can be affected^59,60^. The stomatopods in the present study may have been affected by seawater pH in western waters of Hong Kong which declined significantly by 0.25 units between 2004 and 2016. Temperature is also known to be an important factor affecting crustacean growth and survival^41,61,62^. Specifically, temperature anomalies could influence growth, recruitment and in some cases, survival of marine organisms^63–66^. Unusually elevated water temperatures can deplete dissolved oxygen in hypolimnetic water^67,68^, which in turn suppresses respiration of marine organisms, resulting in their failure in reproduction and stock recruitment, and hence a decrease in total food web biomass^68–70^. Cold-water anomaly has also caused unprecedented mortality to corals and many fish species^71,72^ and reduced local abundance of crab species *Petrolisthes armatus* in Florida in 2010^73^. Higher mean temperatures from June to August of 2015 and more cold days in January to March of 2016 were recorded in Hong Kong compared with the other two study years (2004 and 2013–2014)^74–76^. Specifically, in 2015 Hong Kong had the hottest June since temperatures were first recorded in 1884^77^. This unusual weather condition may have contributed to relatively low abundance and biomass of stomatopods in 2015–2016.

**Figure 6:**
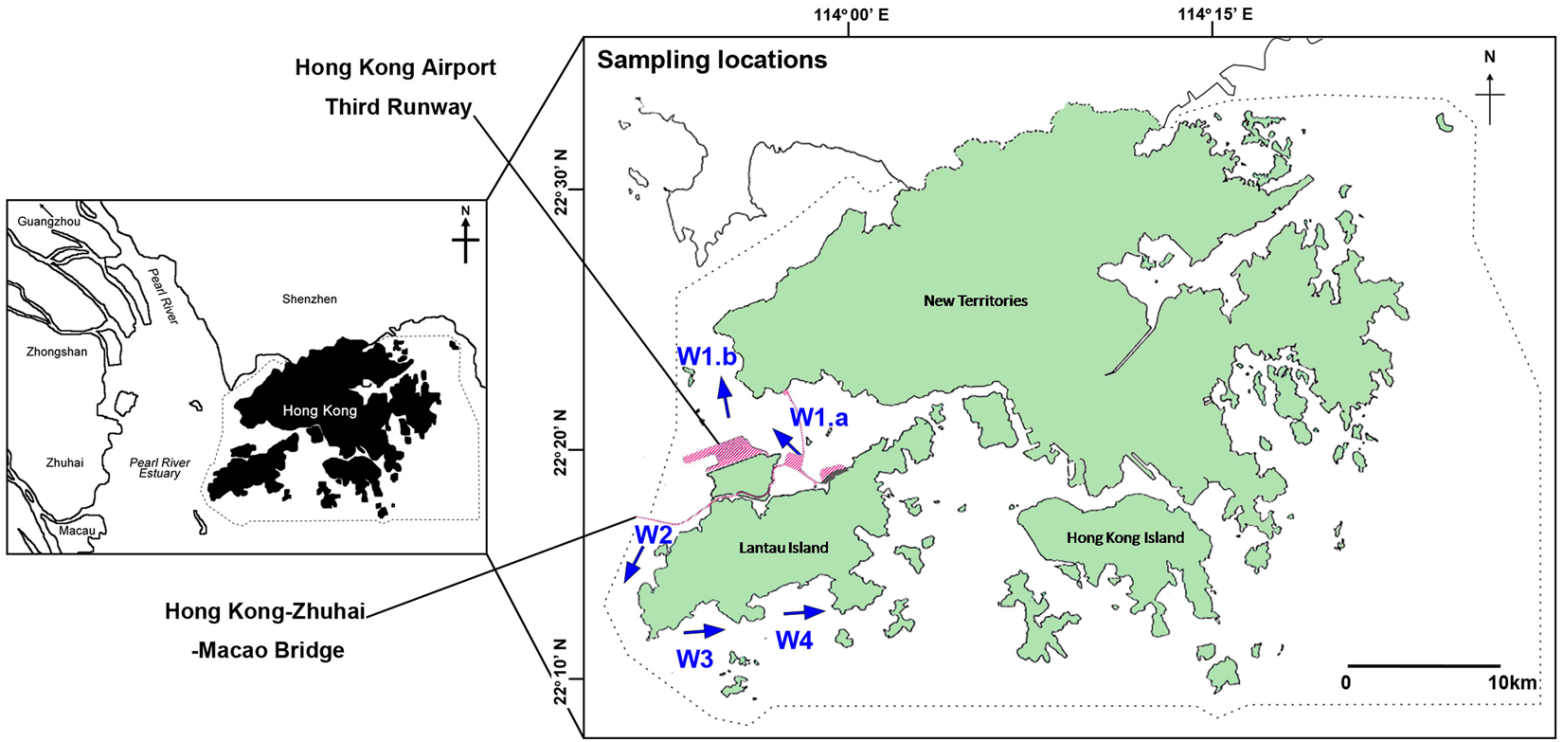
Sampling locations of benthic crustaceans in the western waters of Hong Kong. W1.a, W1.b, W2, W3 and W4 indicate the location of the transects in the present study. The same transacts were surveyed throughout the entire study period, except W1.a which was surveyed only in 2004 (2004; before the trawl ban) and W1.b which was surveyed in June 2013 to May 2014 (2013–2014; immediately after the trawl ban) and June 2015 to May 2016 (2015–2016; 3.5 years after the trawl ban).

Anthropogenic disturbance, such as the ongoing coastal construction projects^78^ (i.e., the Hong Kong-Zhuhai-Macau Bridge and the Third Runway System of Hong Kong International Airport; Fig. 6) and illegal trawling in western waters^79^ (Table S3), may also have contributed to the absence of increases in abundance and biomass of stomatopods. Moreover, the trawl ban policy in Hong Kong waters alone may not be sufficient to reduce the total fishing effort in the local fishery due to the increase in fishing pressure from other miscellaneous gears, especially cage trapping and gill netting, which are legal, non-trawling fishing methods^80–85^ (Table S4) and because enforcement of the ban is limited. This could have limited the anticipated recovery of stomatopods after the trawl ban. Similarly, in the Hawke Box of Canada which is a bottom trawling exclusion zone, the snow crab *Chionoecetes opilio* showed no signs of increase seven years after the implementation of the closure. This was attributed to the redistributed and intensified non-trawling fishing effort on the snow crab inside the Hawke Box^86,87^.

The relatively short time frame of the current study may have also partly contributed to the differences observed in changes in abundance/biomass compared with size-based indicators. Several studies have estimated the duration needed for benthic communities to recover after a trawl ban with estimates ranging from 1.9 to 10 years^88–90^. These studies highlight the variability in the length of time required for ecosystem recovery, as well as of factors such as life history and environmental and fishery conditions. Findings from other survey-based studies also demonstrated that short-term observations may not be sufficient to detect changes in abundance and biomass after implementing management interventions^22^, potentially longer duration may be needed for recovery to be detected^91^. Therefore, long-term monitoring is highly recommended to detect any further improvement of crustacean community from the trawl ban.

## Summary

This study investigated possible outcomes of the trawl ban on the populations of four stomatopod species in the western waters of Hong Kong. Our working hypotheses have been partly supported in relation to some size-based indicators although whether the trawl ban is the main driving cause of the changes could not be determined. However, the hypotheses on abundance, biomass and relative percentage were not supported; several possible confounding factors were identified. Reduction of physical disturbance on benthic substratum coupled with the increase of food availability and substratum heterogeneity brought by the trawl ban could have contributed to the observed increases in mean weight and carapace length for four stomatopod species, as well as proportion of large-sized individuals in their population. However, increases in predatory fishes and crabs in Hong Kong western waters after the trawl ban may impose great predatory and competition pressure on the study species, and subsequently prevent increases in abundance or biomass of all stomatopods. Additionally, other biotic or non-biotic, factors may also mask or prevent a recovery process, such as illegal fishing with trawlers; increased legal, non-trawling fishing efforts; declines in pH; weather abnormalities; ongoing marine construction work in the Pearl River estuary, and insufficient time for recovery at the time of this study in this heavily degraded marine ecosystem.

Our findings suggest the need for a broader, more regional approach for effective coastal fishery management and for a clearer understanding of the several possible confounding factors that would affect the recovery. A wider range of factors such as those discussed in this study should be considered to ensure effective implementation and monitoring of fishery management measures, including, in this case, more effective enforcement of the ban. Longer term study on these stomatopod species, however, will be needed to further assess the mechanisms, pathways and conditions of how the trawl ban could benefit Hong Kong’s fishery resources.

## Materials and Methods

### Study sites and field sampling

Sampling was conducted in the western waters adjacent to Tuen Mun, the Brothers Channel and Lantau Island in Hong Kong (22°12′N–22°20′N, 113°51′E–114°00′E; Fig. 6). The western waters are strongly affected by seasonal freshwater input from the Pearl River^92^. The salinity of western waters varies from 18 psu to 33 psu between summer and winter^74^. The western waters have been under stress from rapid urbanization and industrialization in the Pearl River Delta region^93,94^, and large-scale marine construction activities in the area including the Hong Kong-Zhuhai-Macau Bridge and the Third Runway System of Hong Kong International Airport^78,95–97^.

Three 12-month surveys (once per month throughout the year) each survey along four transects around the western waters of Hong Kong (i.e., transects W1–W4; Fig. 6) were conducted from January 2004 to December 2004 (2004; before the trawl ban), June 2013 to May 2014 (2013–2014; immediately after the trawl ban), and June 2015 to May 2016 (2015–2016; 3.5 years after the trawl ban) to investigate the population structures of the four stomatopod species. It was assumed that the condition in 2004 would represent the situation before the trawl ban and that four transects and ten replicate nets a month were sufficient to produce a representative sample of mantis shrimps for each sampling date^42^. Standardized sampling was conducted during daytime using similar-sized commercial shrimp trawlers throughout the study. A scientific research permit (R1710007) for trawling was granted by the Director of Agriculture, Fisheries and Conservation Department which allowed us to carry out the sampling after the trawl ban.

Along each transect, a trawler (beam size: 2 m; stretched mesh size: 2 cm) with a 15-metre outrigger and ten replicate nets fished for 30 min at a speed of 5–7 km/h, giving a total trawled area of 0.0375–0.0525 km^2^ per transect. We collected all carnivorous crustaceans and predatory fishes from the ten replicate nets in each transect and treated them as one replicate. All samples were immediately sorted on board, stored on ice, and transferred back to the laboratory for identification. Within 48 h of sample collection, samples were identified to species level and counted, and the body weight and carapace length of each specimen measured to the nearest 0.01 g and 1 mm following the method described in Ahyong^30^, respectively. Standardized abundance (ind./km^2^) and biomass (kg/km^2^) in each transect for each survey were subsequently calculated using the Equation 1:

1 $$\begin{array}{c}{\rm{Standardized}}\,{\rm{abundance}}\,{\rm{and}}\,{\rm{biomass}}={\rm{total}}\,{\rm{number}}\,{\rm{or}}\,{\rm{weight}}/\\ \,({\rm{beam}}\,{\rm{width}}\,\ast \,{\rm{number}}\,{\rm{of}}\,{\rm{nets}}\,\ast \,{\rm{boat}}\,{\rm{speed}}\,\ast \,{\rm{time}})\end{array}$$

where beam width was 2 m for each net; number of nets was 10; and boat speed (km/h) and time (h) was recorded during each trawl by a hand-held GPS device.

All experiments were conducted in accordance with relevant guidelines and regulations.

### Data analysis

#### Environmental data

Marine environmental variables (Table S5), which may influence recovery of benthic communities^98,99^, were extracted from the database of Environmental Protection Department (EPD) of the Hong Kong Special Administrative Region Government^74^. The marine bottom water variables for each of the four survey transects during the corresponding surveyed months were used for further data analysis. Due to limited marine sediment data from EPD, the percentage mud data from adjacent areas of the four survey transects, which were collected two times each year, were extracted for further analysis. To explore yearly differences in environmental variables, one-way ANOVA was applied using transects as replicates.

#### Cohort analysis

Cohort analysis was conducted for each of the four stomatopod species to examine their population structures. Individuals from the four monthly transects were combined and their carapace length data were grouped into 2 mm size classes to generate monthly size-frequency histograms. Relative abundance of each size class, which were obtained by dividing the abundance data of that particular size class by the total abundance in that month, was used instead of absolute abundance to reduce the bias from monthly variability in each sampling year^27,100^. Individual cohorts were identified and calculated using the Bhattacharya’s method by Electronic Length Frequency Analysis (ELEFAN; International Center for Living Aquatic Resources Management, Philippines) in FiSAT II (FAO-ICLARM Fish Stock Assessment Tools, Version 1.2.2, Italy). A separation index (SI) of a value of 2 was used as the cut-off value to segregate different cohorts^101^. Means and standard deviations of the carapace length data were obtained for estimating cohort-specific growth rates^100^. For each year, the growth rate of each cohort of each species was estimated by linear regression of carapace length against time. One-way analysis of variance (ANOVA) was used to compare growth rates of each species among years using different cohorts as replicates.

Munro’s growth performance index (ϕ′)^102^ was calculated to compare the growth potential of different species (Equation 2):

2 $${\rm{\varphi }}^{\prime} ={\mathrm{Log}}_{10}{\rm{K}}+2/3\,{\mathrm{Log}}_{10}{{\rm{L}}}_{\infty }$$

where L_∞_ is the asymptotic carapace length; K is the von Bertalanffy growth function (VBGF) growth constant. Both parameters were calculated by the K-Scan function in ELEFAN.

#### Temporal comparison of abundance/biomass

The abundance and biomass, averaging all surveyed months in a year, of the four stomatopod species were compared among years by one-way ANOVA using transects as replicates. To determine the contribution of these four species to all stomatopods and commercially important crustaceans in the catch, all other stomatopods and commercially important decapods, such as shrimps and crabs in each trawl sample, were also identified to species level, weighed and counted.

To test whether stomatopods responded to the trawl ban more quickly than carnivorous crabs, the relative percentage of stomatopods was calculated as the following (Equations 3 and 4):

3 $${\rm{A}} \% ={{\rm{A}}}_{{\rm{stomatopods}}}/{\rm{total}}\,{{\rm{A}}}_{{\rm{carnivorous}}{\rm{crustaceans}}}$$

4 $${\rm{B}} \% ={{\rm{B}}}_{{\rm{stomatopods}}}/{\rm{total}}\,{{\rm{B}}}_{{\rm{carnivorous}}{\rm{crustaceans}}}$$

where A is the abundance, B is the biomass; carnivorous crustaceans include all stomatopods and the carnivorous crabs with a relatively long life span (Table S6). The monthly abundance and biomass of all stomatopods, carnivorous crabs, predatory fishes and relative abundance/biomass of stomatopods were compared by using one-way ANOVA.

#### The relationship between bottom water properties and abundance/biomass

Linear models were used to investigate the relationships between environmental factors, such as suspended solids (SS), dissolved oxygen (DO), chlorophyll-a concentration (Chl-a), bottom water temperature (Temp) and pH, and abundance and biomass of all stomatopods. Logarithmic (natural) transformations were applied on abundance and biomass of all stomatopods to normalize the data. The model was considered as the following (Equation 5):

5 $$\mathrm{Ln}({\rm{A}}\,{\rm{or}}\,{\rm{B}})={{\rm{b}}}_{0}+{{\rm{b}}}_{{\rm{ss}}}{{\rm{X}}}_{{\rm{ss}}}+{{\rm{b}}}_{{\rm{DO}}}{{\rm{X}}}_{{\rm{DO}}}+{{\rm{b}}}_{\text{Chl}-{\rm{a}}}{{\rm{X}}}_{\text{Chl}-{\rm{a}}}+{{\rm{b}}}_{{\rm{Temp}}}{{\rm{X}}}_{{\rm{Temp}}}+{{\rm{b}}}_{{\rm{pH}}}{{\rm{X}}}_{{\rm{pH}}}+{\varepsilon }_{{\rm{i}}}$$

where A or B is the abundance or biomass of all stomatopods; Xss, X_DO_, X_Chl-a_, X_Temp_, and X_pH_ are the values of the environmental factors SS, DO, Chl-a, Temp and pH; bss, b_DO_, b_Chl-a_, b_Temp_ and b_pH_ are regression coefficients for the five environmental factors. b_0_ is the intercept, and *ε*_i_ is the error associated with the measurement.

#### Temporal comparison of size-based indicators

Yearly differences of size-based indicators (i.e., mean individual weight, mean carapace length and L_95%_) of each species were investigated by one-way ANOVA. L_95%_ represents upper 95% quartiles of the carapace length and is recommended as an alternative to maximum observed length (L_max_) and asymptotic length (L_∞_) which are strongly influenced by sample size^43^.

Three size classes (small, medium and large) were defined according to the maximum carapace length recorded for each stomatopod species (Table S7). For each species, the relative abundance (or biomass) of each size class in each year was calculated as the abundance (or biomass) of that size class over its total abundance (or biomass).

#### Size spectrum

Size spectra, which take into account the size distribution, abundance and biomass of a species in the community, are sensitive to fishing pressure, with the slope of the size-based linear model decreasing with increasing fishing mortality^43,103^. Hence, size spectrum can be used to assess the effect of fishing on a population^104^. In general, a shallow slope of a size spectrum indicates a relatively higher abundance (or biomass) of large size classes of the target species in the community^43,58^. The size spectrum of a fish community is typically dome-shaped^105^. Quadratic regression is, therefore, commonly applied to model the dome-shaped size spectrum and the curvature of the size spectrum generally decreases with increasing fishing mortality^57^. As dome-shaped size spectra were observed in the populations of the four stomatopod species in this study, the quadratic regression model

6 $${{\rm{y}}}_{{\rm{i}}}={\rm{a}}+{{\rm{bx}}}_{i}+{{{\rm{cx}}}_{{\rm{i}}}}^{2}$$

was utilized. An increase in curvature after the trawl ban would indicate an increase in the relative abundance/biomass of large size class individuals.

To well-fit the regression curve, stomatopod abundance and biomass data were separated into 5 mm carapace length classes. Size spectra were constructed by plotting natural logarithm of the abundance/biomass of each size class versus the median size of each length class^55,106^. The curvature value was computed from the model as the coefficient c in the quadratic equation.

All statistical analyses were performed in FiSAT II (FISAT II - FAO-ICLARM Stock Assessment Tool, Version 1.2.2, Italy) and R 3.1.2 (2014).

### Experiments including animals

The trawling survey conducted in the present study was funded by a Research Grants Council of the Government of the Hong Kong Special Administrative Region (HKSAR) via a Collaborative Research Fund (CRF Project No. HKU5/CRF/12 G) to KMYL for the purposes of investigating the effects of the trawl ban. Agriculture, Fisheries and Conservation Department of HKSAR has granted us a scientific research permit (R1710007) to conduct all the trawl surveys.

## Electronic supplementary material

Supplementary figures and tables

## Data Availability

The datasets generated during and/or analyzed during the current study are available from the corresponding author on reasonable request.
