# Supplementary material for: Trawl ban in a heavily exploited marine environment: Responses in population dynamics of four stomatopod species
Source: Sci Rep. 2018 Dec 14;8:17876. doi: 10.1038/s41598-018-35804-7 (PMC6294824; doi:10.1038/s41598-018-35804-7)
Supplement: Supplementary file 1 — Supplementary figures and tables [file 41598_2018_35804_MOESM1_ESM.docx]

**Trawl ban in a heavily exploited marine environment: Responses in population dynamics of four stomatopod species**

Lily S. R. Tao, Karen K. Y. Lui, Edward T. C. Lau, Kevin K. Y. Ho, Yanny K. Y. Mak, Yvonne Sadovy de Mitcheson, Kenneth M. Y. Leung*****

*The Swire Institute of Marine Science and School of Biological Sciences, The University of Hong Kong, Pokfulam, Hong Kong, China*

*Corresponding author: Kenneth M. Y. Leung

E-mail: [kmyleung@hku.hk](mailto:shirutao@hku.hk)

Tel.: +852 2299 0607

Fax: +852 2517 6082


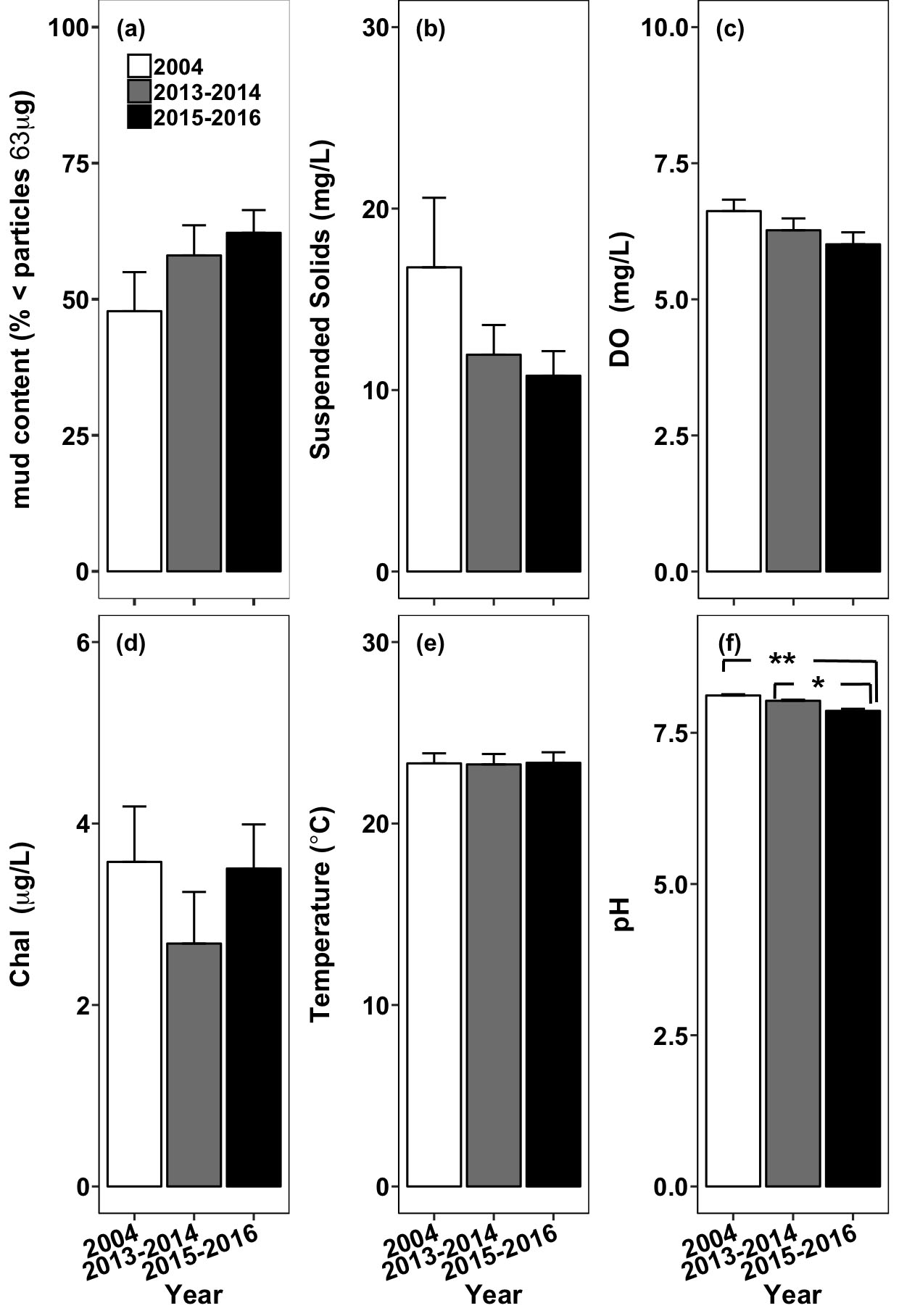


**Figure S1**. (a) Percentage mud content (mean + SE; % < 63 μm), (b) Suspended Solids (mean + SE; mg/L), (c) Dissolved Oxygen (DO; mean + SE; mg/L), (d) Chlorophyll-a (Chl a; mean + SE; μg/L), (e) Bottom water temperature (mean + SE; ℃) and (f) pH collected in the western waters of Hong Kong from January 2004 to December 2004 (2004; before the trawl ban), June 2013 to May 2014 (2013–2014; immediately after the trawl ban), and June 2015 to May 2016 (2015–2016; 3.5 years after the trawl ban). Significant differences are indicated by asterisks: * p<0.05; ** p<0.01, *** p<0.001.


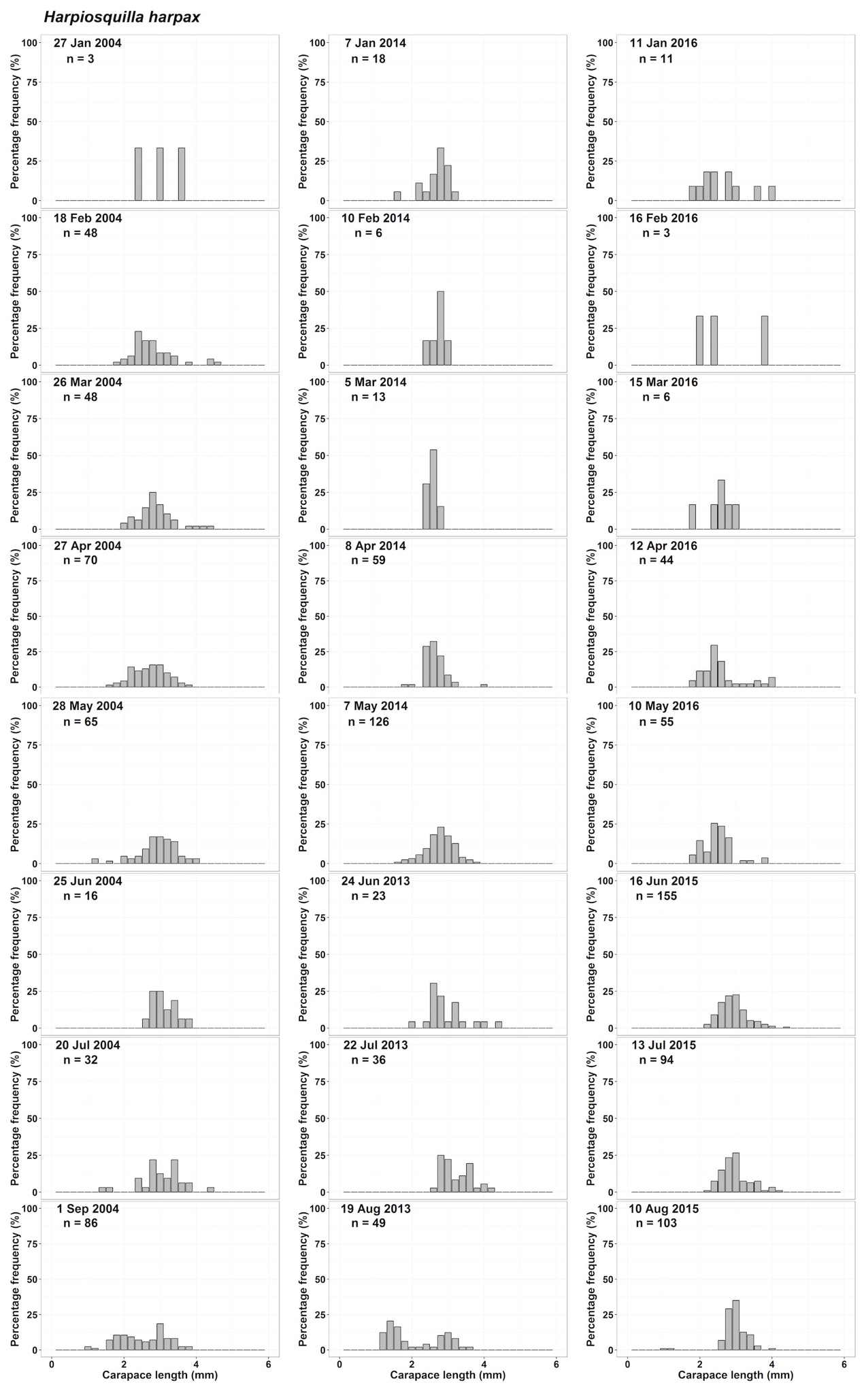


**Figure S2**. (Con’t)


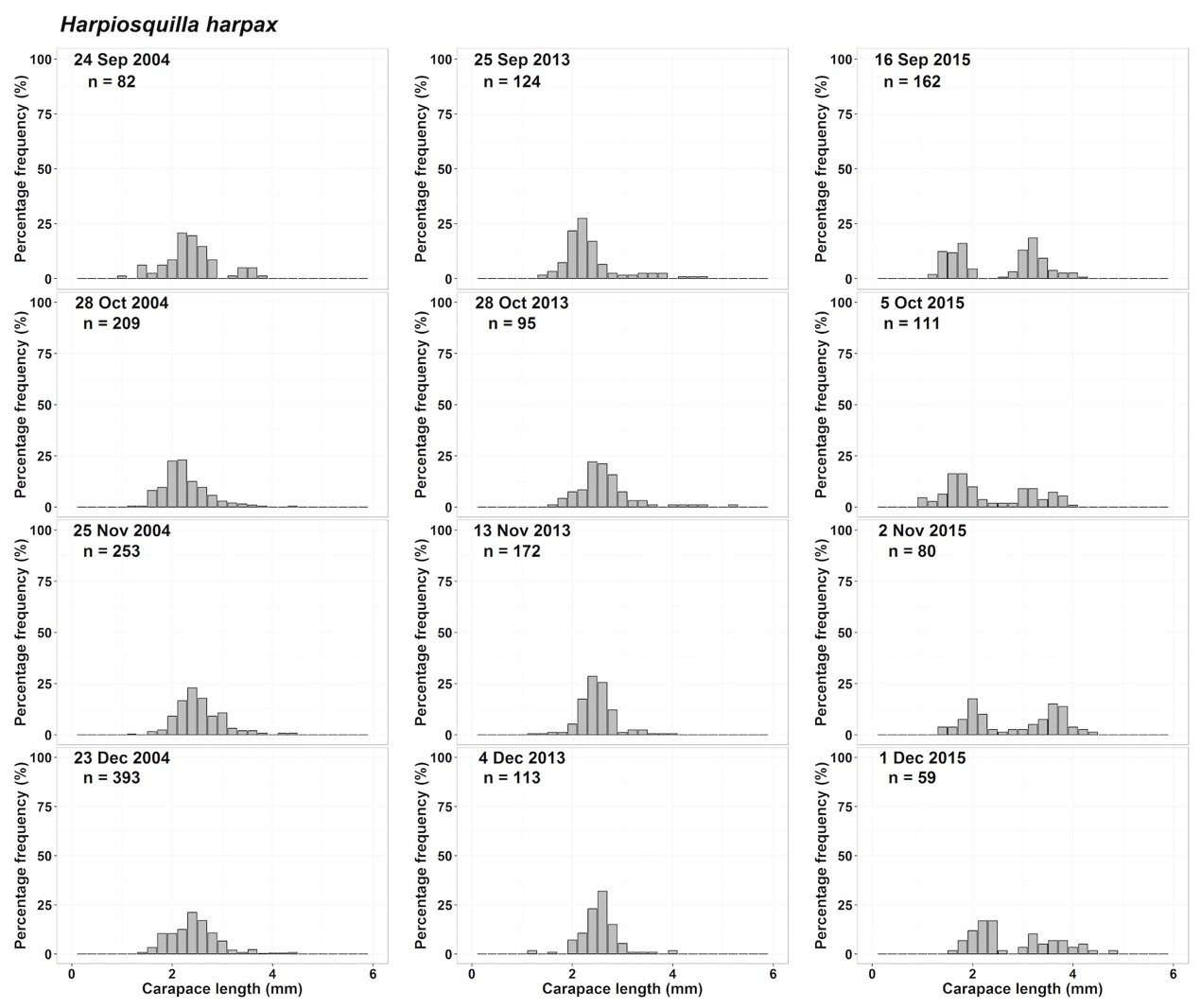


**Figure S2.** *Harpiosquilla harpax* Monthly size-frequency distribution at the western waters from January 2004 to December 2004 (2004; before trawling ban), June 2013 to May 2014 (2013–2014; immediately after the trawl ban), and June 2015 to May 2016 (2015–2016; 3.5 years after the trawl ban). n: number of individuals sampled; note that the second and third columns are arranged by months for ease of comparison.


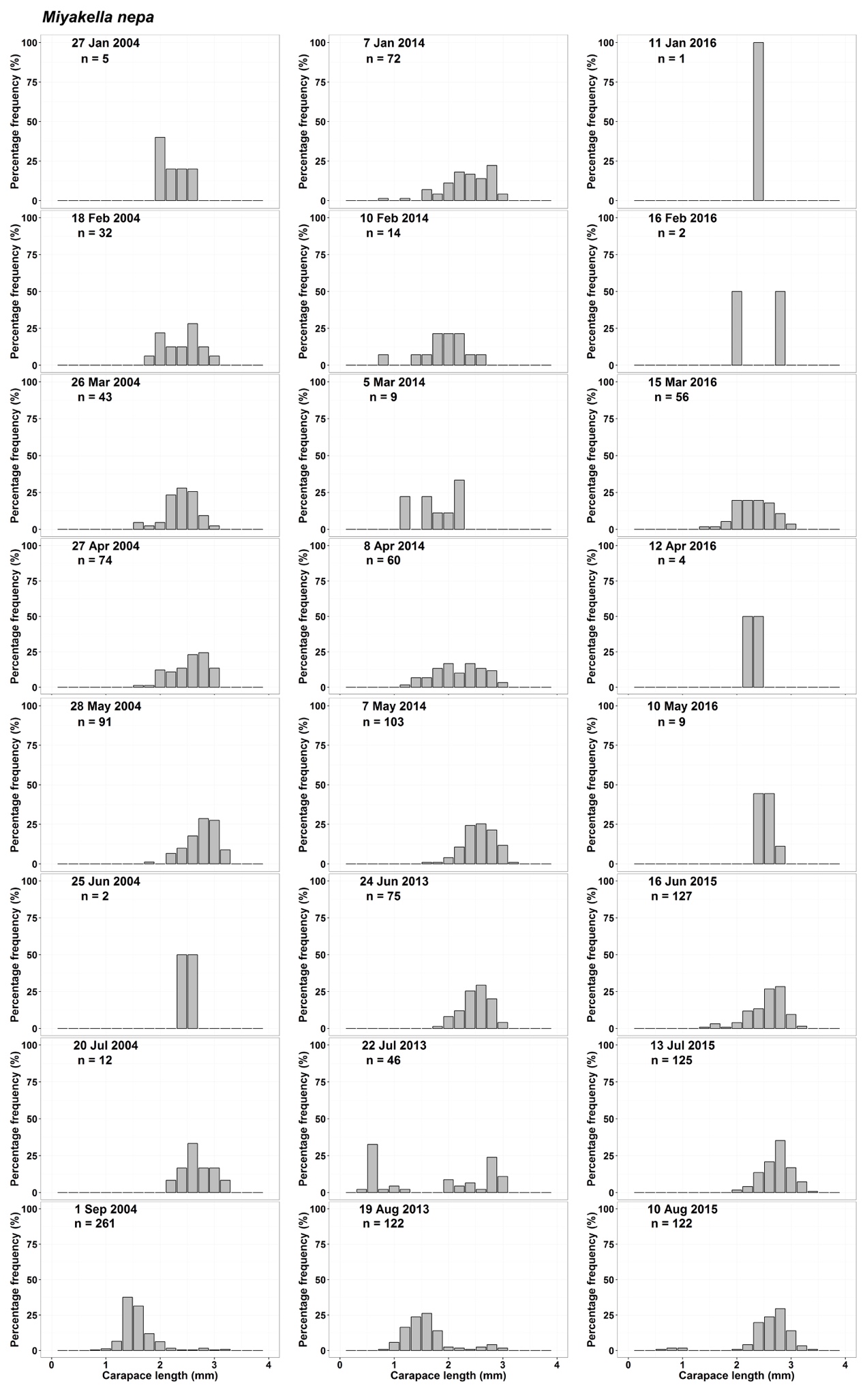


**Figure S3.** (Con’t)


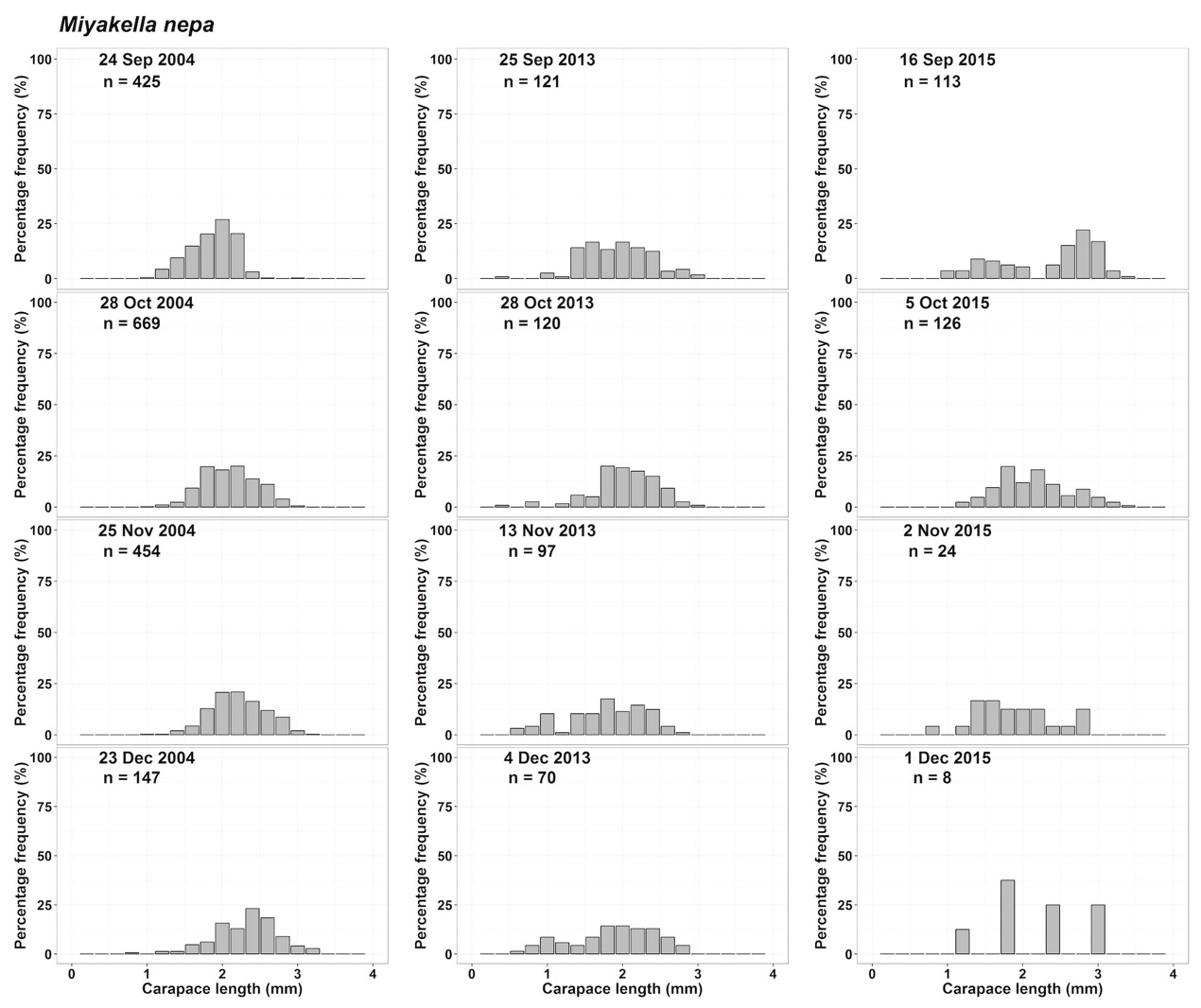


**Figure S3**. *Miyakea nepa* Monthly size-frequency distribution at the western waters from January 2004 to December 2004 (2004; before trawling ban), June 2013 to May 2014 (2013–2014; immediately after the trawl ban), and June 2015 to May 2016 (2015–2016; 3.5 years after the trawl ban). n: number of individuals sampled; note that the second and third columns are arranged by months for ease of comparison.


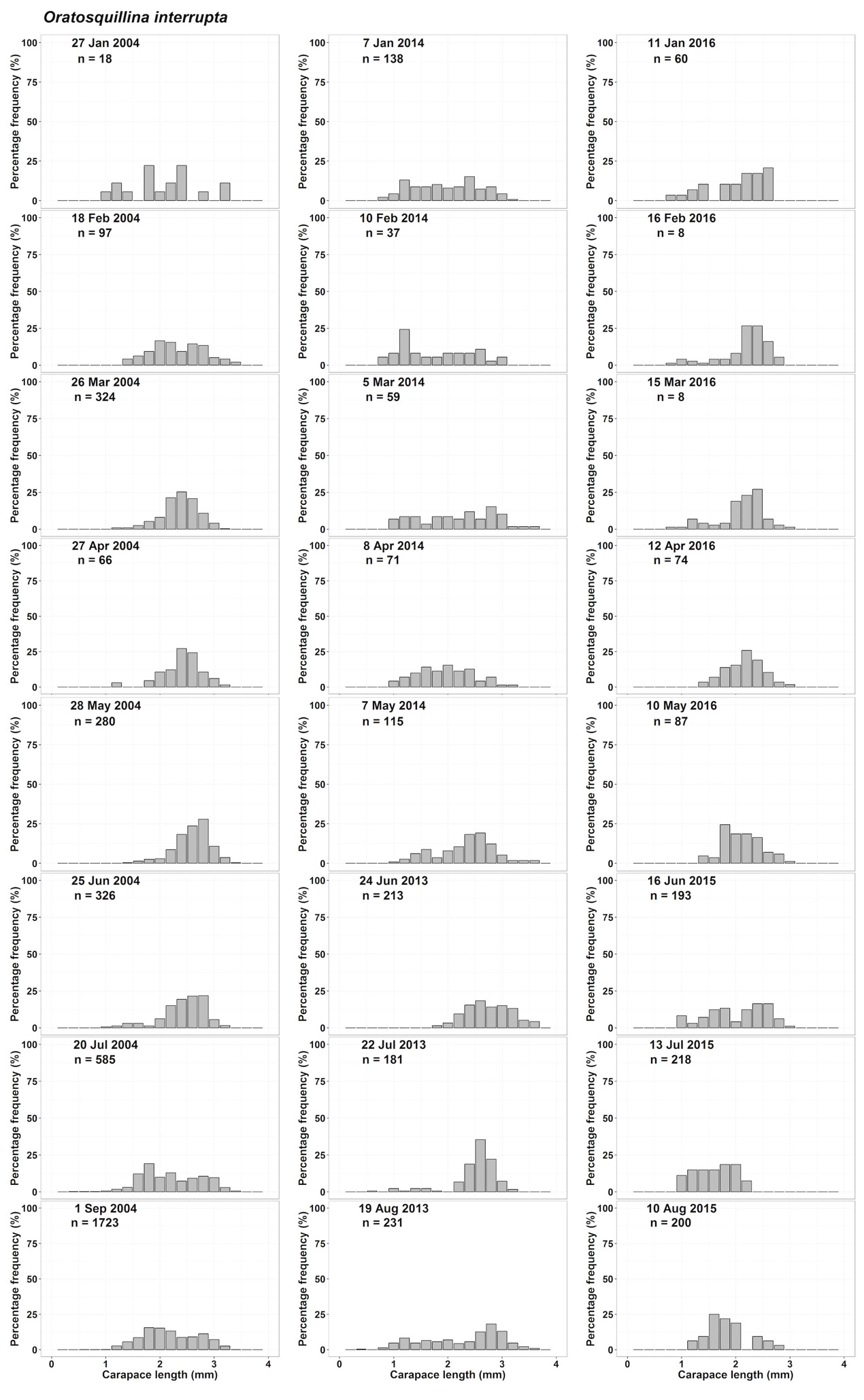


**Figure S4.** (Con’t)

**
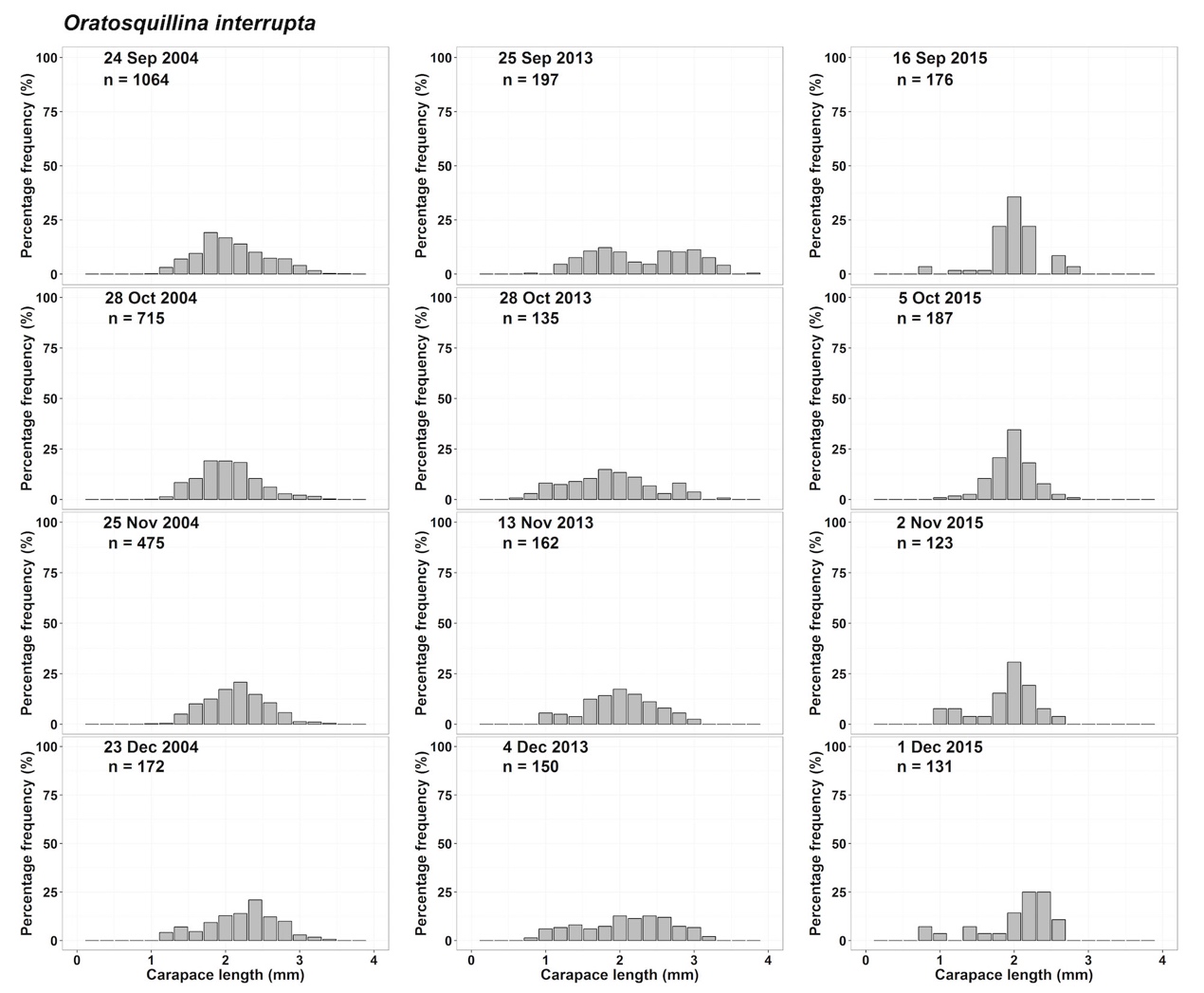
**

**Figure S4.** *Oratosquillina interrupta* Monthly size-frequency distribution at the western waters from January 2004 to December 2004 (2004; before trawling ban), June 2013 to May 2014 (2013–2014; immediately after the trawl ban), and June 2015 to May 2016 (2015–2016; 3.5 years after the trawl ban). n: number of individuals sampled; note that the second and third columns are arranged by months for ease of comparison.


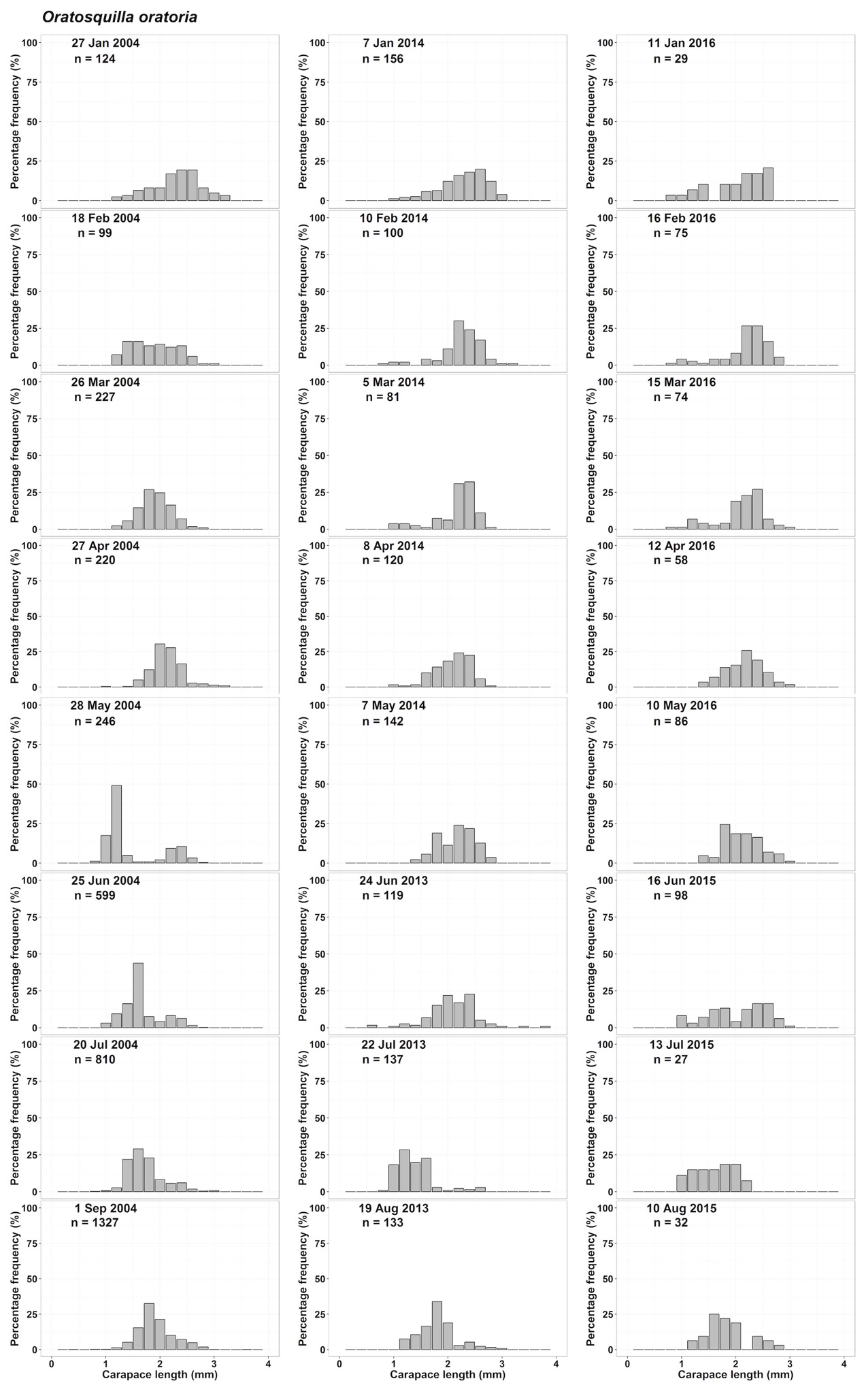


**Figure S5.** (Con’t)


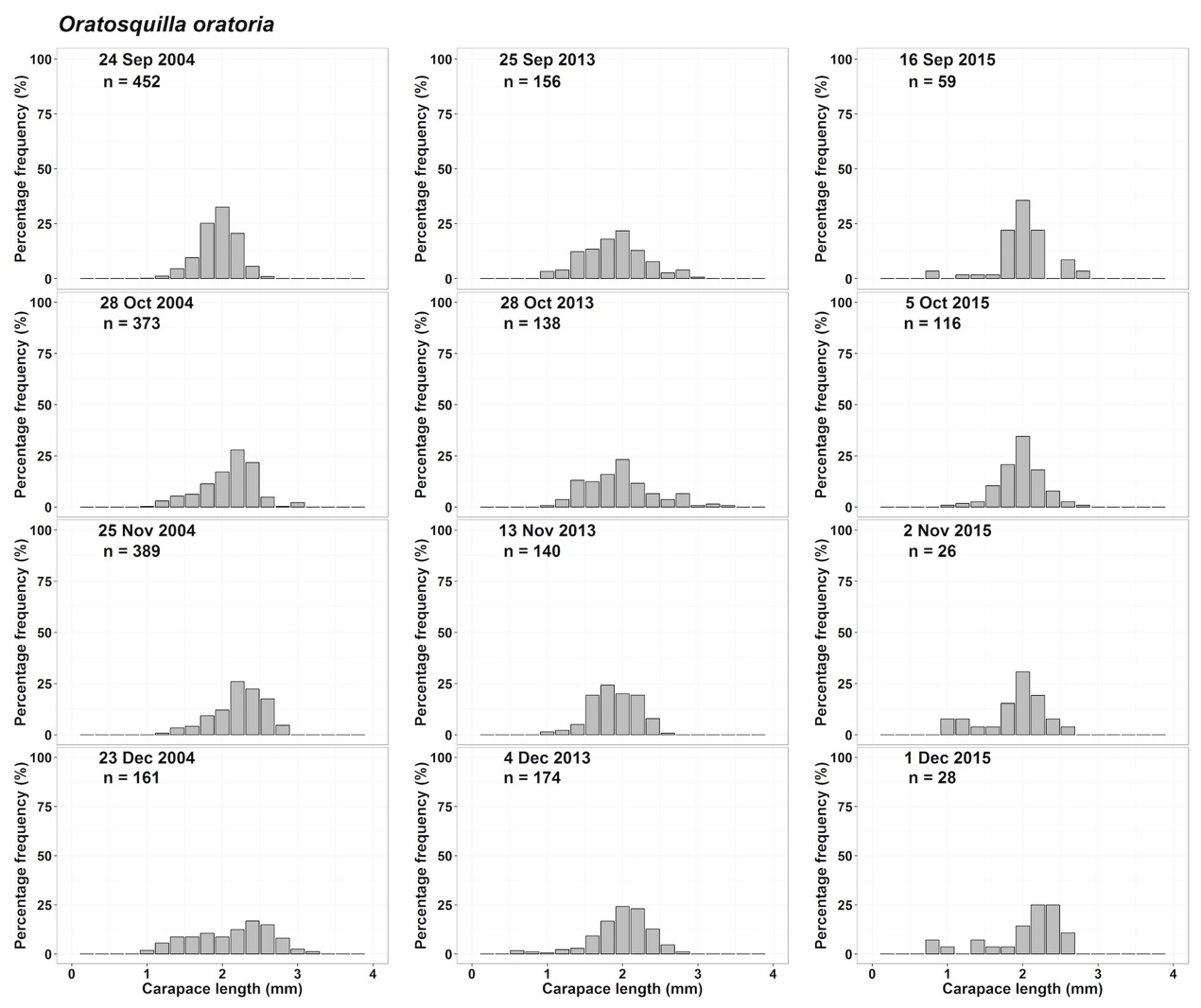


**Figure S5.** *Oratosquilla oratoria* Monthly size-frequency distribution at the western waters from January 2004 to December 2004 (2004; before trawling ban), June 2013 to May 2014 (2013–2014; immediately after the trawl ban), and June 2015 to May 2016 (2015–2016; 3.5 years after the trawl ban). n: number of individuals sampled; note that the second and third columns are arranged by months for ease of comparison.


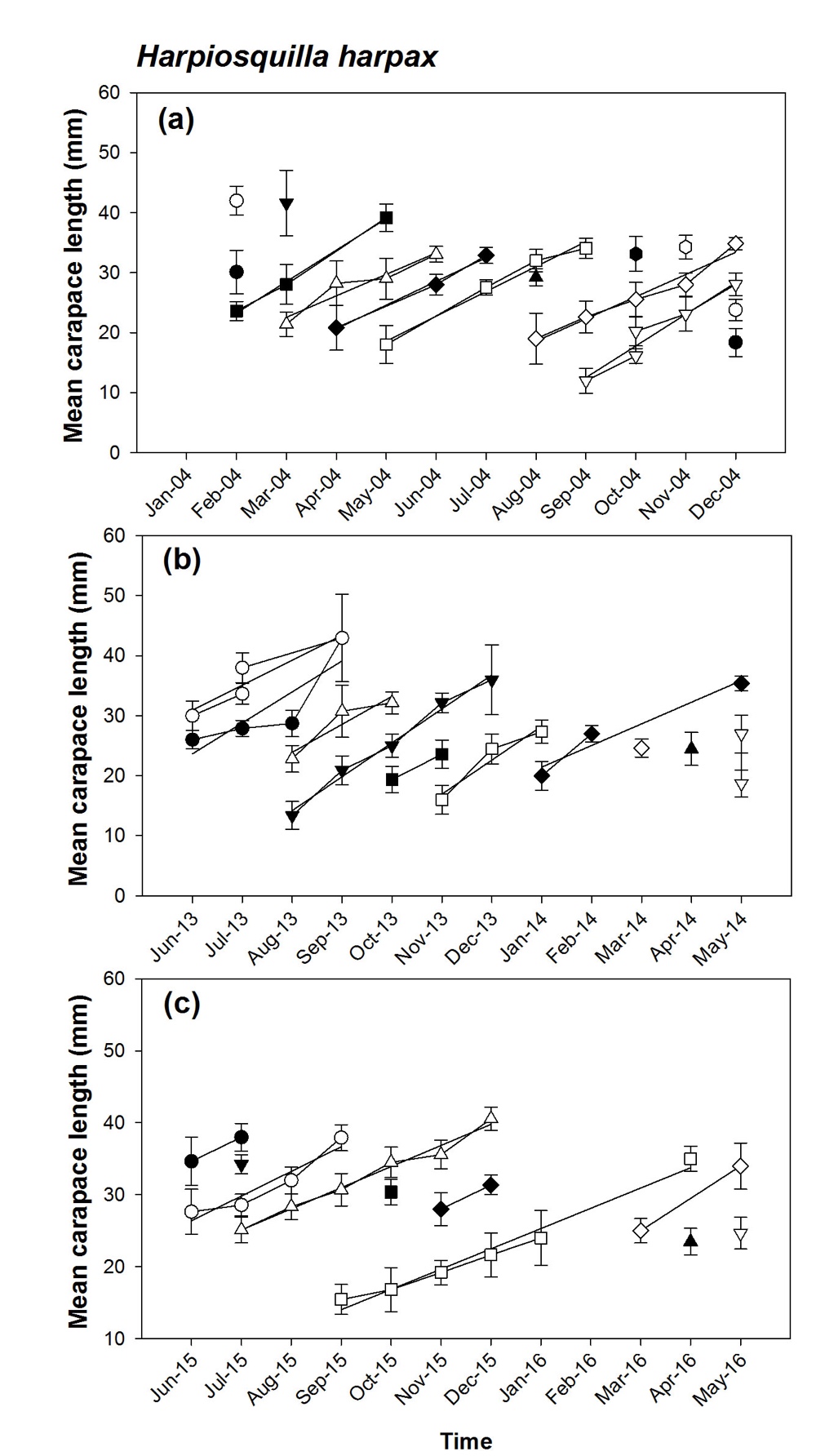


**Figure S6.** *Harpiosquilla harpax*. Estimated growth (mean ± SD) of different cohorts at the western waters from: (a) January 2004 to December 2004 (2004; before trawling ban), (b) June 2013 to May 2014 (2013–2014; immediately after the trawl ban), and (c) June 2015 to May 2016 (2015–2016; 3.5 years after the trawl ban).


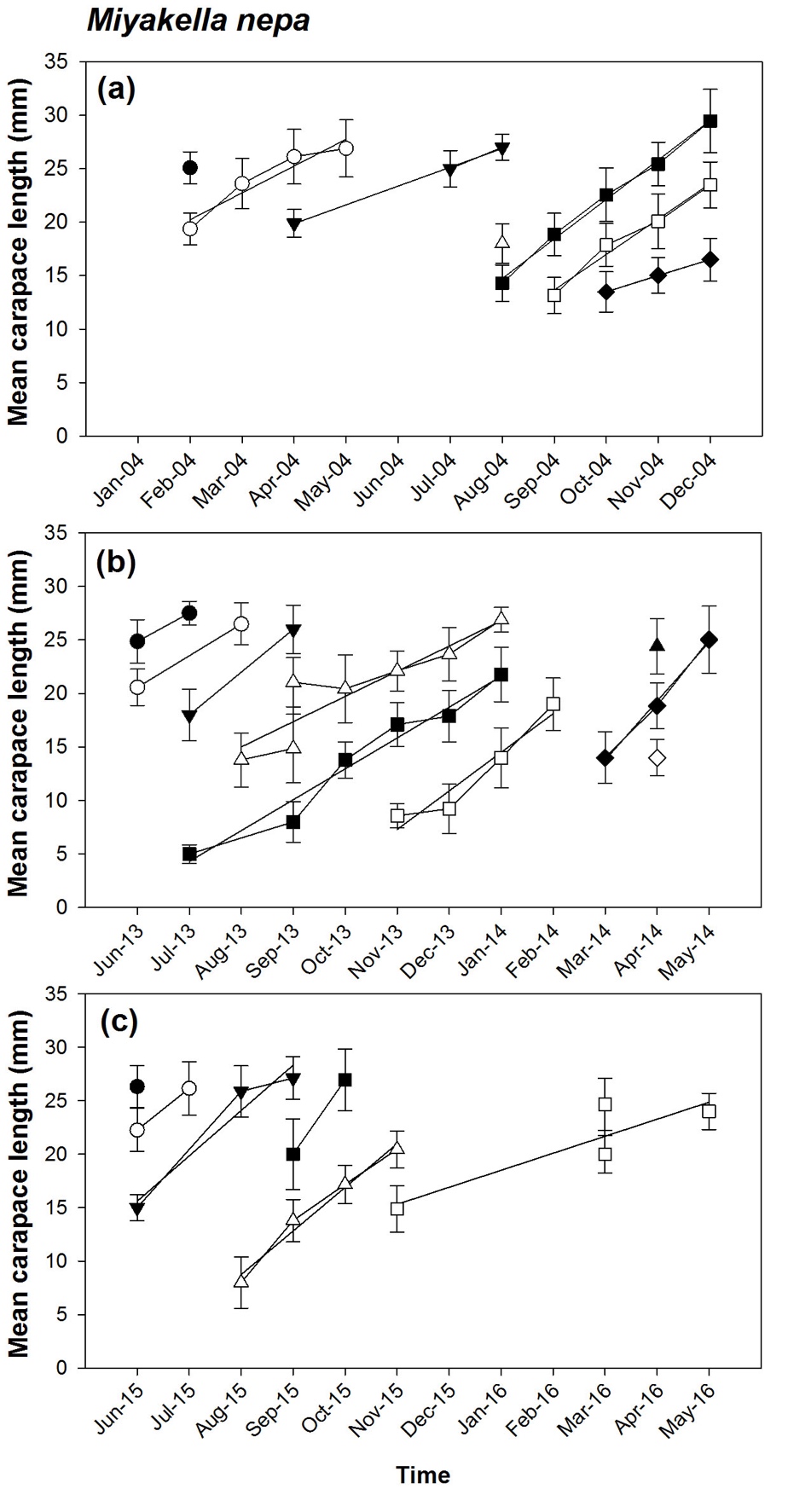


**Figure S7.** *Miyakella nepa*. Estimated growth (mean ± SD) of different cohorts at the western waters from (a) January 2004 to December 2004 (2004; before trawling ban), (b) June 2013 to May 2014 (2013–2014; immediately after the trawl ban), and (c) June 2015 to May 2016 (2015–2016; 3.5 years after the trawl ban).


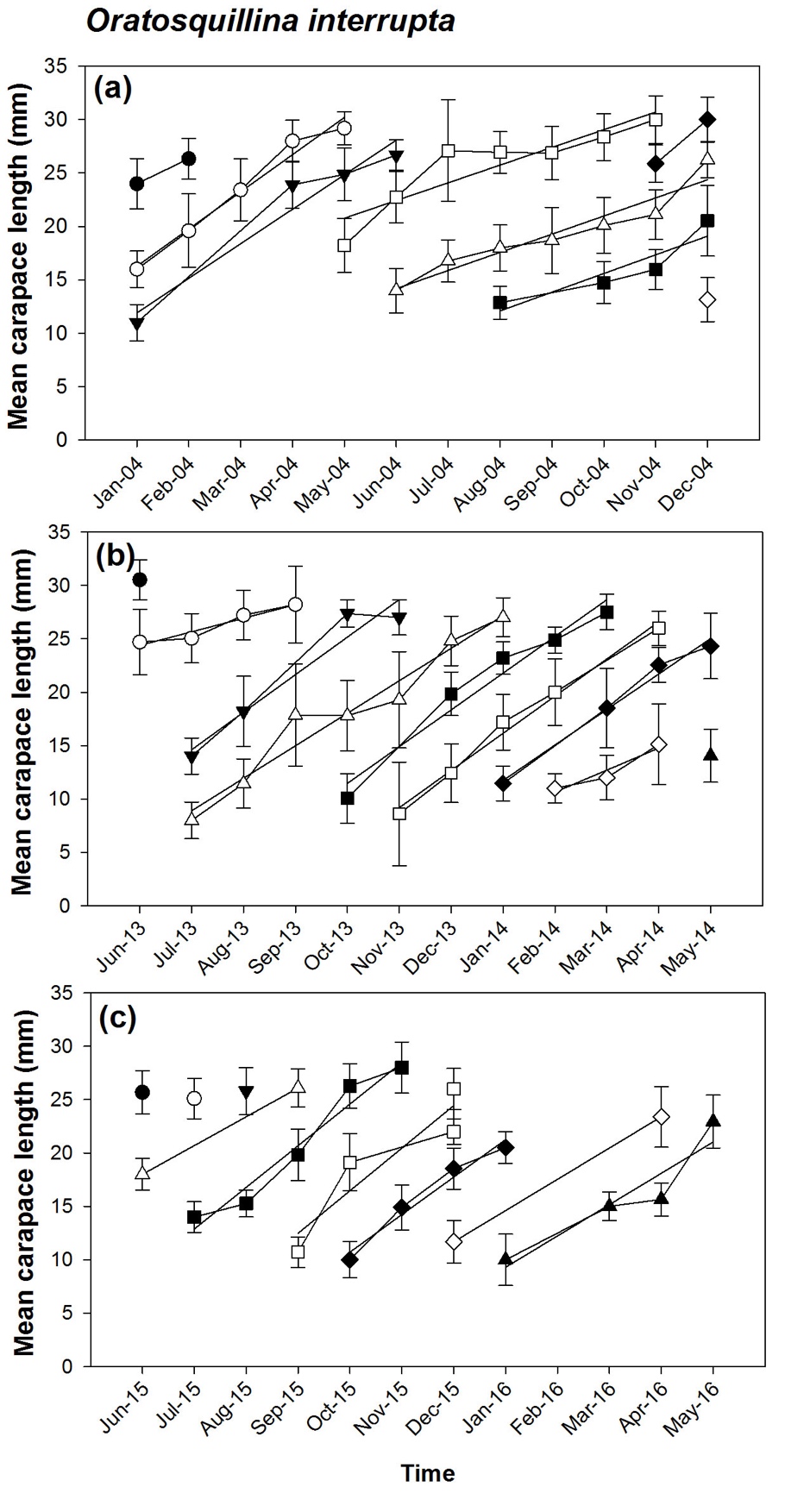


**Figure S8.** *Oratosquillina interrupta*. Estimated growth (mean ± SD) of different cohorts at the western waters from (a) January 2004 to December 2004 (2004; before trawling ban), (b) June 2013 to May 2014 (2013–2014; immediately after the trawl ban), and (c) June 2015 to May 2016 (2015–2016; 3.5 years after the trawl ban).


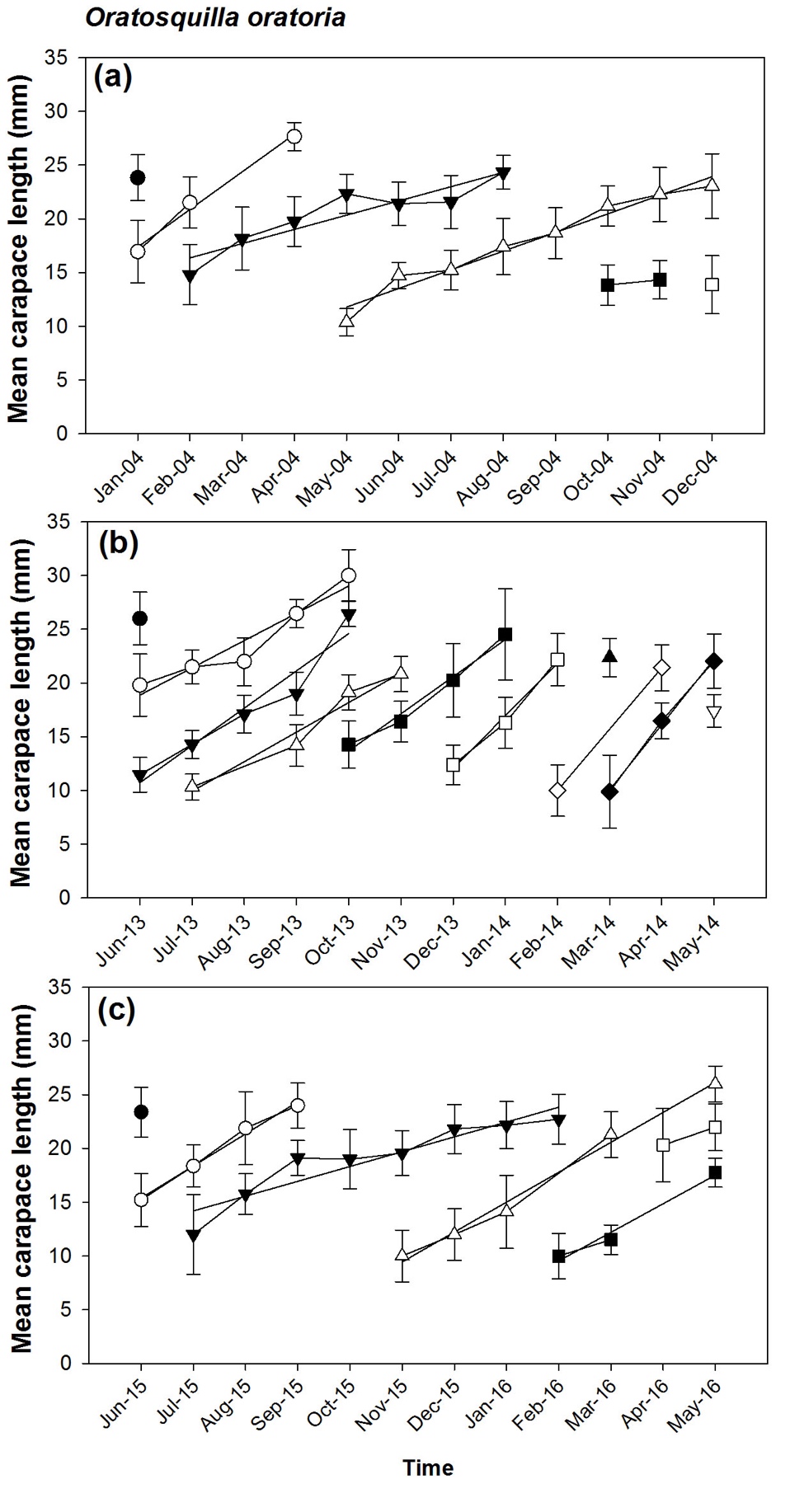


**Figure S9.** *Oratosquilla oratoria*. Estimated growth (mean ± SD) of different cohorts at the western waters from (a) January 2004 to December 2004 (2004; before trawling ban), (b) June 2013 to May 2014 (2013–2014; immediately after the trawl ban), and (c) June 2015 to May 2016 (2015–2016; 3.5 years after the trawl ban).


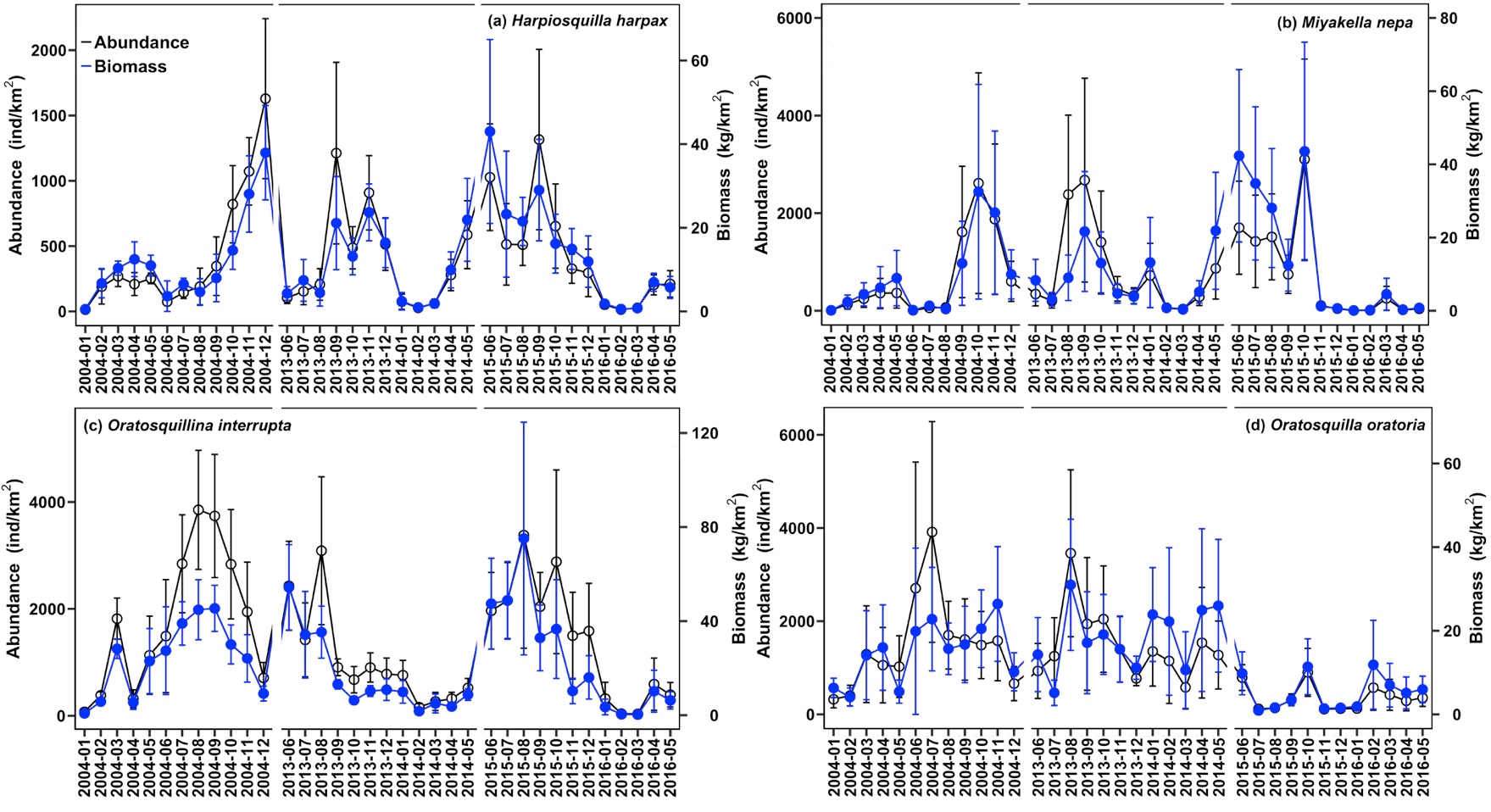


**Figure S10.** Monthly abundance (Mean ± SEM; individuals/km^2^) and biomass (Mean ± SEM; kg/km^2^) of the four investigated stomatopod species: (a) *Harpiosquilla harpax*, (b) *Miyakella nepa*, (c) *Oratosquillina interrupta*, and (d) *Oratosquilla oratoria,* collected in western waters from January 2004 to December 2004 (2004; before trawl ban), June 2013 to May 2014 (2013–2014; immediately after the trawl ban), June 2015 to May 2016 (2015–2016; 3.5 years after the trawl ban). Note that both y-axes have different scales.


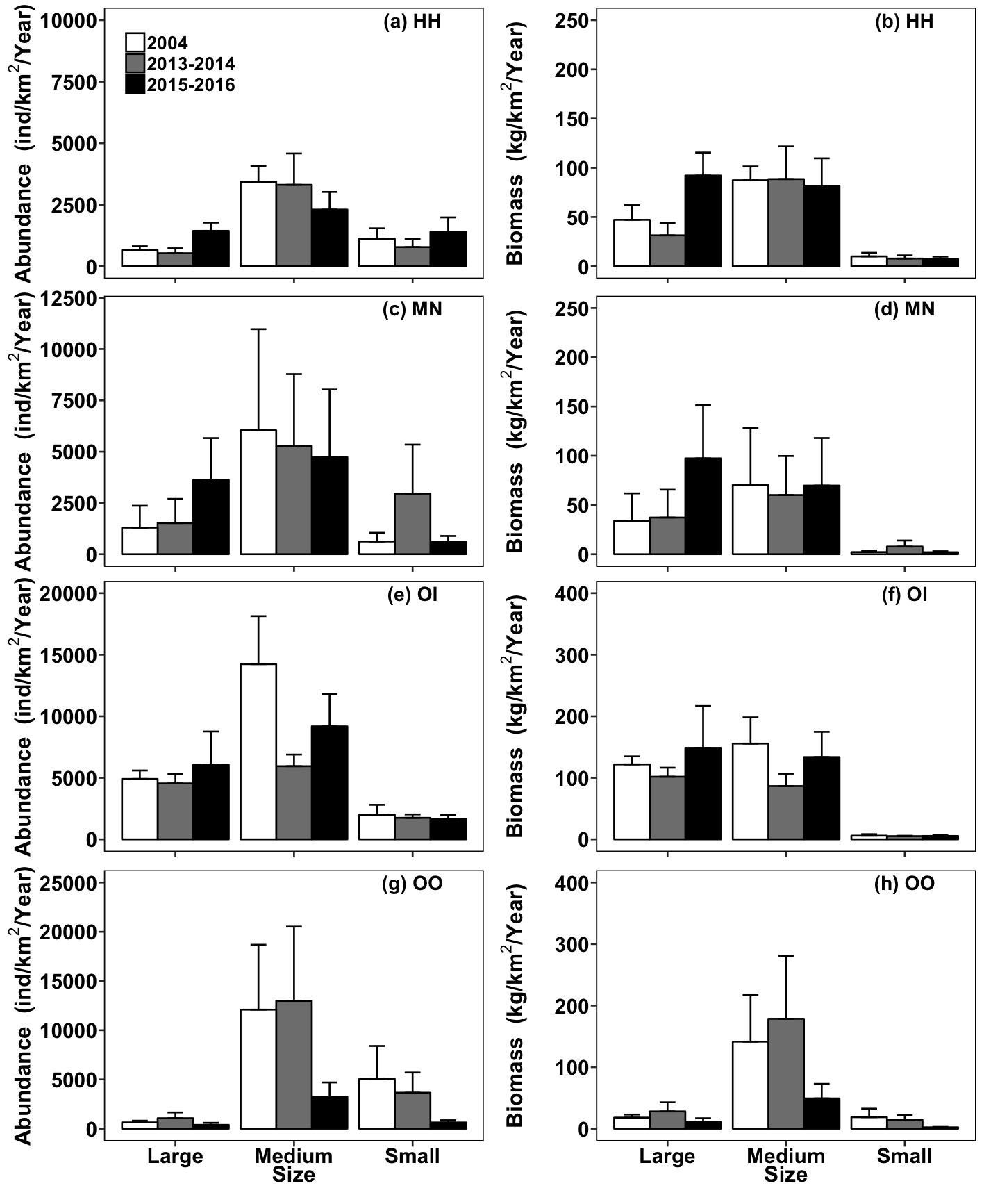


**Figure S11.** Abundance (mean + SE; ind/km^2^/Year) and biomass (mean + SE; kg/km^2^/Year) of three size classes for *Harpiosquilla harpax* (HH; a-b), *Miyakella nepa* (MN; c-d), *Oratosquillina interrupta* (OI; e-f) and *Oratosquilla oratoria* (OO; g-h) collected in western waters from 2004, 2013-2014 and 2015-2016.


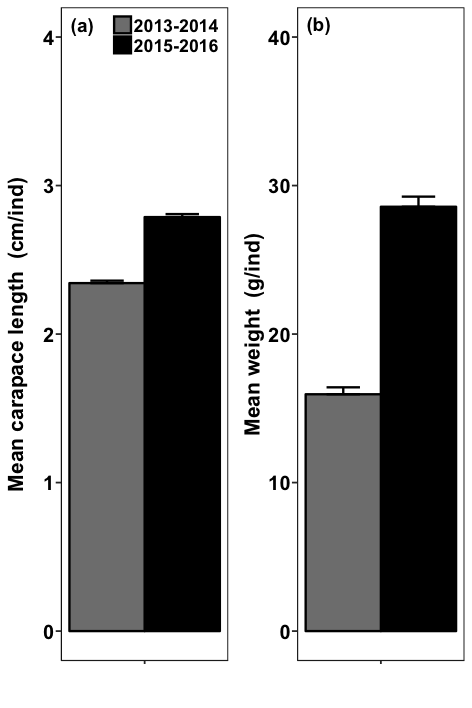


**Figure S12.** (a) Mean carapace length (mean ± SE; cm/ind) and (b) mean weight (mean ± SE; g/ind) of crab assemblage collected at Outer estuary (WO) from June 2013 to May 2014 (2013–2014; immediately after the trawl ban), June 2015 to May 2016 (2015–2016; 3.5 years after the trawl ban).


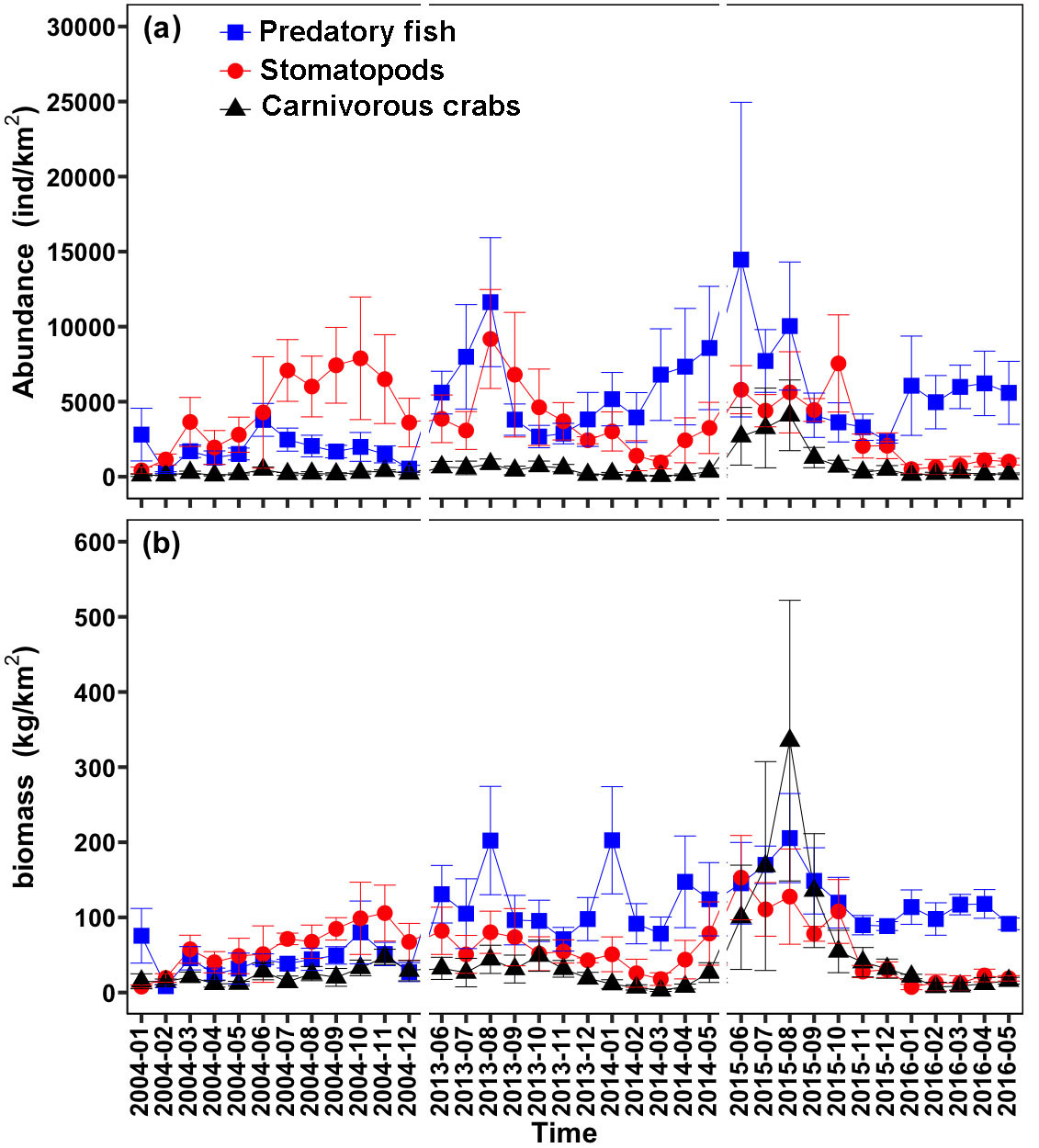


**Figure S13.** (a) Monthly abundance (Mean ± SEM; individuals/km^2^) and (b) biomass (Mean ± SEM; kg/km^2^) of the predatory fishes, all stomatopods and carnivorous crabs collected in western waters from January 2004 to December 2004 (2004; before trawl ban), June 2013 to May 2014 (2013–2014; immediately after the trawl ban), June 2015 to May 2016 (2015–2016; 3.5 years after the trawl ban).

**Table S1.** Commercial crustaceans used in this study.

| Order | Family | Species |
| --- | --- | --- |
| Decapoda | Portunidae | *Charybdis feriatus* |
| Decapoda | Portunidae | *Portunus pelagicus/trituberculatus* |
| Decapoda | Portunidae | *Portunus sanguinolentus* |
| Decapoda | Portunidae | *Scylla serrata* |
| Decapoda | Penaeidae | *Metapenaeopsis sp.* |
| Decapoda | Penaeidae | *Metapenaeus affinis* |
| Decapoda | Penaeidae | *Metapenaeus joyneri* |
| Decapoda | Penaeidae | *Metapenaeus moyebi* |
| Decapoda | Penaeidae | *Parapenaeopsis hardwickii* |
| Decapoda | Penaeidae | *Parapenaeopsis hungerfordi* |
| Decapoda | Penaeidae | *Parapenaeopsis tenella* |
| Decapoda | Penaeidae | *Penaeus latisulcatus* |
| Decapoda | Penaeidae | *Penaeus merguiensis* |
| Decapoda | Penaeidae | *Penaeus monodon* |
| Decapoda | Penaeidae | *Penaeus penicillatus* |
| Decapoda | Penaeidae | *Penaeus semisulcayus* |
| Decapoda | Penaeidae | *Trachysalambria sp.* |
| Decapoda | Solenoceridae | Solenocera crassicornis |
| Stomatopoda | Squillidae | *Anchisquilla fasciata* |
| Stomatopoda | Squillidae | *Carinosquilla multicarinata* |
| Stomatopoda | Squillidae | *Clorida decorata* |
| Stomatopoda | Squillidae | *Dictyosquilla foveolata* |
| Stomatopoda | Squillidae | *Erugosquilla woodmasoni* |
| Stomatopoda | Squillidae | *Harpiosquilla harpax* |
| Stomatopoda | Squillidae | *Miyakella nepa* |
| Stomatopoda | Squillidae | *Oratosquilla oratoria* |
| Stomatopoda | Squillidae | *Oratosquillina interrupta* |

**Table S2.** The relationship between environmental factors and stomatopods’ abundance (a) and biomass (b).

| (a) Abundance | *p* < 0.001, Adjusted R^2^= 0.275 | | | |
| --- | --- | --- | --- | --- |
|  | Estimate | Std. Error | t value | *p* value |
| (Intercept) | -309.358 | 93.366 | -3.313 | 0.001 |
| Temperature (℃) | 4.068 | 0.781 | 5.209 | < 0.001 |
| Suspended Solids (mg/L) | 0.044 | 0.130 | 0.338 | 0.736 |
| pH | 31.672 | 12.468 | 2.540 | 0.012 |
| Dissolved Oxygen (mg/L) | 1.946 | 2.209 | 0.881 | 0.380 |
| Chlorophyll-a (μg/L) | 0.135 | 0.722 | 0.186 | 0.852 |
|  |  |  |  |  |
| (b) Biomass | *p* < 0.001, Adjusted R^2^= 0.24 | | | |
|  | Estimate | Std. Error | t value | *p* value |
| (Intercept) | -10.677 | 3.743 | -2.853 | 0.005 |
| Temperature (℃) | 0.114 | 0.031 | 3.643 | < 0.001 |
| Suspended Solids (mg/L) | 0.002 | 0.005 | 0.432 | 0.666 |
| pH | 1.492 | 0.500 | 2.986 | 0.003 |
| Dissolved Oxygen (mg/L) | -0.085 | 0.089 | -0.961 | 0.338 |
| Chlorophyll-a (μg/L) | 0.027 | 0.029 | 0.940 | 0.349 |

**Table S3.** Number of successful prosecution against illegal fishing activities by AFCD from 2013-2017^64^.

|  | 2013 | | 2014 | | 2015 | | 2016 | | 2017 | |
| --- | --- | --- | --- | --- | --- | --- | --- | --- | --- | --- |
|  | Illegal trawling | Other illegal fishing activities | Illegal trawling | Other illegal fishing activities | Illegal trawling | Other illegal fishing activities | Illegal trawling | Other illegal fishing activities | Illegal trawling | Other illegal fishing activities |
| Number of successful prosecution | 13 | 7 | 11 | 7 | 3 | 36 | 3 | 11 | 0 | 3 |

**Table S4.** Number of different vessels before and after trawl-ban.

Source: Agriculture Fisheries and Conservation Department (2011-2016)^65-70^

| **Type of vessel** | 2010 | 2011 | 2012 | 2013 | 2014 | 2015 |
| --- | --- | --- | --- | --- | --- | --- |
| Pair Trawler | 582 | 630 | 617 | 598 | 526 | 561 |
| Stern Trawler | 156 | 160 | 149 | 152 | 133 | 120 |
| Shrimp Trawler | 349 | 376 | 352 | 350 | 315 | 261 |
| Hang Trawler | 43 | 43 | 42 | 42 | 38 | 40 |
| Gill Netter | 237 | 221 | 202 | 164 | 161 | 246 |
| Long Liner | 118 | 119 | 113 | 65 | 68 | 113 |
| Hand Liner | 64 | 60 | 55 | 22 | 22 | 43 |
| Purse Seiner | 93 | 92 | 90 | 71 | 73 | 93 |
| Miscellaneous | 2260 | 2325 | 2372 | 2524 | 3203 | 3569 |

**Table S5.** Selected environmental variables (abbreviation; Unit; marine water or sediment variable) for environmental analysis.

| Bottom water temperature (Temp; ℃; marine water variable) | pH (marine water variable) |
| --- | --- |
| Chlorophyll-a (Chl a:μg/L; marine water variable) | Suspended Solids (SS; mg/L; marine water variable) |
| Dissolved Oxygen (DO; mg/L; marine water variable) | The percentage mud (TPM; fraction <63 μm; marine water variable) |

**Table S6.** Carnivorous crabs used in this study.

| Species names | Longevity | Reference |
| --- | --- | --- |
| *Charybdis feriatus* | 3 years | Dash *et al*. 2014 |
| *Portunus trituberculatus* | around 3 years | Xue *et al*. 1996 |
|  | around 3 years | Sugilar *et al.* 2012 |
| *Portunus pelagicus* | 3 years | Dineshbabu *et al*. 2008 |
|  | 2.5 years | Sukumaran *et al*. 1997 |
| *Portunus sanguinolentus* | 2.5 years | Dineshbabu *et al*. 2007 |
|  | 2.5 years | Sukumaran *et al*. 1997 |
| *Scylla serrata* | 3- to 4-years | Bonine *et al*. 2008 |

**References**:

Bonine, K.M., Bjorkstedt, E.P., Ewel, K.C. & Palik, M. Population characteristics of the mangrove crab *Scylla serrata* (Decapoda: Portunidae) in Kosrae, Federated States of Micronesia: effects of harvest and implications for management. *Pac. Sci.* **62,** 1-19 (2008).

Dash, G., *et al.* (2014) Analysis of fishery and stock of the portunid crab, *Charybdis feriata* (Linnaeus, 1758) from Veraval waters, north-west coast of India. *Indian J. Fish.* **61,** 1-9 (2014).

Dineshbabu, A., Sreedhara, B. & Muniyappa, Y. Fishery and stock assessment of *Portunus sanguinolentus* (Herbst) from south Karnataka coast, India. J.Mar.Biol.Ass.India, **49**, 134-140 (2007).

Dineshbabu, A.P., Sreedhara, B. & Muniyappa, Y. Biology and exploitation of the blue swimmer crab, *Portunus pelagicus* (Linnaeus, 1758), from south Karnataka coast, India. *Indian J. Fish.* **55,** 215-220 (2008).

Sugilar, H., Park, Y.C., Lee, N.H., Han, D.W. & Han, K.N. Population dynamics of the swimming crab *Portunus trituberculatus* (Miers, 1876)(Brachyura, Portunidae) from the West Sea of Korea. *International Journal of Oceanography and Marine Ecological System,* **1,** 36-49 (2012).

Sukumaran, K. & Neelakantan, B. Food and feeding of *Portunus (Portunus) sanguinolentus* (Herbst) and *Portunus (Portunus) pelagicus* (Linnaeus) (Brachyura: Portunidae) along Karnataka coast. *Indian J. Mar. Sci.* **26,** 35-38 (1997).

Xue, J.Z., Du N.S., Lai, W. & Wu, H.X. A review of studies on *Portunus trituberculatus* in China. *Donghai Marine Science,* **15,** 60-65 (1996).

**Table S7.** Size classes defined by carapace length for each stomatopod species.

| Species | Small | Medium | Large |
| --- | --- | --- | --- |
| *Harpiosquilla harpax* | ≤ 2 cm | > 2 cm and ≤ 3 cm | ≥ 3 cm and ≤ 5.5 cm |
| *Miyakea nepa* | ≤ 1 cm | > 1 cm and ≤ 2 cm | ≥ 2 cm and ≤ 3.5 cm |
| *Oratosquillina interrupta* | ≤ 1 cm | > 1 cm and ≤ 2 cm | ≥ 2 cm and ≤ 3.5 cm |
| *Oratosquilla oratoria* | ≤ 1 cm | > 1 cm and ≤ 2 cm | ≥ 2 cm and ≤ 3.5 cm |
